# A Unified Framework for Modeling Continuum and Rarefied Gas Flows

**DOI:** 10.1038/s41598-017-13274-7

**Published:** 2017-10-12

**Authors:** Hong Xiao, Ke Tang

**Affiliations:** 1 School of Power and Energy, Northwestern Polytechnical University, Xi’an, 710072 China; 2 Department of Engineering, University of Cambridge, Cambridge, CB2 1PZ UK

## Abstract

The momentum and heat transport in rarefied gas flows is known to deviate from the classical laws of Navier and Fourier in Navier-Stokes-Fourier (NSF) equations. A more sophisticated Nonlinear Coupled Constitutive Model (NCCM) has been derived from the Boltzmann equation to describe gaseous and thermal transport both in continuum and rarefied gas flows. We first develop a unified numerical framework for modeling continuum and rarefied flows based on the NCCM model both in two and three dimensions. Special treatment is given to the complex highly nonlinear transport equations for non-conserved variables that arise from the high degree of thermal nonequilibrium. For verification and validation, we apply the present scheme to a stiff problem of hypersonic gas flows around a 2D cylinder, a 3D sphere, and the Apollo configuration both in continuum and rarefied situations. The results show that the present unified framework yields solutions that are in better agreement with the benchmark and experimental data than are the NSF results in all studied cases of rarefied problems. Good agreement is observed between the present study and the NSF results for continuum cases. The results show that this study provides a unified framework for modeling continuum and rarefied gas flows.

## Introduction

The numerical study of continuum-rarefied gas flows is of great interest since it can provide fundamental knowledge about flow physics and provide a theoretical tool to precisely predict the aerodynamic or aerothermodynamic performance of hypersonic vehicles and/or Micro-Electro-Mechanical Systems^1–4^, *et al*. The Navier-Stokes-Fourier (NSF) equations have serious limitations in capturing the correct flow physics under high nonequilibrium conditions such as for rarefied gas^5^. Although using Direct Simulation Monte Carlo (DSMC)^6^ is common in the investigation of rarefied gas flows, it has unacceptable memory demands in near continuum states because it uses simulation molecules to model the movement of molecules and the collision of gas flows. Therefore, the development of a unified scheme for continuum-rarefied gas flows is a challenge in the field of computational fluid dynamics.

In the NSF equations, non-conserved variables associated with thermal nonequilibrium, such as the shear stress tensor and heat flux vector, are described in conjunction with the linear constitutive relations of the gradients of velocity and temperature. Note that these classical relations are derived from the assumption of near local thermal equilibrium^7^. However, this assumption is no longer applicable in nonequilibrium gas flows because of the lack of molecular collisions^8^. To remove this shortcoming of the near-local-thermal-equilibrium approach, generalized hydrodynamic equations were derived by Eu^9^. An important result in Eu’s work is that the transport equations for non-conserved variables, such as stresses and heat flux, are derived from the Boltzmann equation in consideration of positive entropy generation. Subsequently, these transport equations for non-conserved variables were simplified by removing the high-order term and unsteady term, then named Nonlinear Coupled Constitutive Relations (NCCR)^10,11^. This simplification was based on the assumption that the relaxation times of the non-conserved variables such as stress and heat flux are very short in comparison with conserved variables such as the density and velocity. This NCCR model has been successfully applied to some challenging problems of nonequilibrium gas flows where the NSF equations have been found to be inappropriate^12,13^. In the course of these endeavours, we found that this simplification sometimes might cause non-physical oscillation and result in non-convergence in numerical iteration. Differ from the previous work^12,13^, the objective of this study is to develop a unified framework for modeling continuum and rarefied gas flows without simplifications namely to NCCM modelí.

## Unified Framework: Nonlinear Coupled Constitutive Model

The Boltzmann-Curtiss equation for diatomic molecular with a moment of inertia *I* and an angular momentum *j* can be expressed under the assumption of no external force,

1 $$[\frac{\partial }{\partial t}+{\bf{v}}\cdot \nabla +\frac{j}{I}\frac{\partial }{\partial \psi }]\,f({\bf{v}},{\bf{r}},t)=C[f]$$

where the term *C*[*f*] represents the collision integral of the interaction among the particles. Boltzmann-Curtiss equation is irreversiable and consequently expected to describe macroscopic processes progressing irreversible toward equilibrium. This feature is more useful form by means of the entropy generation.

The balance equations for mass, momentum, and energy can be derived by differentiating the statistical formulas of the three quantities with time and then substituting Boltzmann-Curtiss equation. They do not have contributions from Boltzmann collision integral since the three quantities are conserved variables whose molecular expressions are the collisional invariants of Boltzmann collision integral. However, the balance equations contain nonconserved variables, such as, the shear stress **Π**, the heat flux **Q**, and the excess normal stress Δ, whose molecular expressions do not yield a collisional invariant. We refer to them as nonconserved variables or nonconserved moments. To derive the evolution equations for nonconserved variables, we can use the velocity moment and its differential equation. The two important issues involved in this procedure are the definition of distribution function and the treatment of Boltzmann collision integral. In the present study, the distribution function evolves as functional of macroscopic moments, but the flux dependence of distribution function is strictly dictated by entropy production. The treatment of Boltzmann collision integral described in supplementary material is built on the nonlinear cumulant approximation of dissipation terms. The procedure of deriving the constitutive equations from the kinetic equation is also described in the previous works^9^. Finally, the conservation laws and the constitutive equations can be expressed in a compact form:

2 $$\begin{array}{l}\rho \frac{D}{Dt}\,\,[\begin{array}{l}{[1,\rho {\bf{u}},\rho E]}^{T}/\rho \\ {\boldsymbol{\Pi }}/\rho \\ ({\boldsymbol{\Pi }}+{\rm{\Delta }}{\bf{I}})/\rho \\ {\bf{Q}}/\rho \end{array}]+\nabla \cdot [\begin{array}{l}{[{\bf{u}},p{\bf{I}}+{\boldsymbol{\Pi }}+{\rm{\Delta }}{\bf{I}},(p{\bf{I}}+{\boldsymbol{\Pi }}+{\rm{\Delta }}{\bf{I}})\cdot {\bf{u}}+{\bf{Q}}]}^{T}\\ {{\boldsymbol{\Psi }}}^{({\boldsymbol{\Pi }})}\\ {{\boldsymbol{\Psi }}}^{({\rm{\Delta }})}\\ {{\boldsymbol{\Psi }}}^{({\bf{Q}})}\end{array}]\\ \quad =\,[\begin{array}{l}{[0,0,0]}^{T}\\ {Z}^{({\boldsymbol{\Pi }})}+{{\rm{\Lambda }}}^{({\boldsymbol{\Pi }})}\\ {Z}^{({\rm{\Delta }})}+{{\rm{\Lambda }}}^{({\rm{\Delta }})}\\ {Z}^{({\bf{Q}})}+{{\rm{\Lambda }}}^{({\bf{Q}})}\end{array}]\end{array}$$

In this expression, *ρ* is the mass density, **u** is the fluid velocity, *p* is the pressure, *E* is the total energy density and *T* is the gas temperature. *Z*, Λ and ***Ψ*** represent the kinematic term, the dissipative term and the high-order flux term, respectively:

3 $$\begin{array}{l}{{\rm{\Lambda }}}^{({\boldsymbol{\Pi }})}=\langle m{[{\bf{cc}}]}^{\mathrm{(2)}}\,C[f]\rangle ,\,{{\rm{\Lambda }}}^{({\rm{\Delta }})}=\langle (\frac{1}{3}m{c}^{2}-p/n)\,C[f]\rangle ,\\ {{\rm{\Lambda }}}^{({\bf{Q}})}=\langle (\frac{1}{2}m{c}^{2}+{H}_{rot}-m\hat{h})\,{\bf{c}}C[f]\rangle ,\,{{\boldsymbol{\Psi }}}^{({\boldsymbol{\Pi }})}=\langle m{[{\bf{c}}{\bf{c}}]}^{\mathrm{(2)}}\,{\bf{c}}f\rangle ,\\ {{\boldsymbol{\Psi }}}^{({\rm{\Delta }})}=\langle (\frac{1}{3}m{c}^{2}-p/n)\,{\bf{c}}f\rangle ,\,{{\boldsymbol{\Psi }}}^{({\bf{Q}})}=\langle (\frac{1}{2}m{c}^{2}+{H}_{rot}-m\hat{h})\,{\bf{cc}}f\rangle ,\\ {Z}^{({\boldsymbol{\Pi }})}=\langle f(\frac{D}{Dt}+{\bf{c}}\cdot \nabla +\frac{j}{I}\frac{\partial }{\partial \psi })\,m{[{\bf{cc}}]}^{\mathrm{(2)}}\rangle ,\\ {Z}^{({\rm{\Delta }})}=\langle f(\frac{D}{Dt}+{\bf{c}}\cdot \nabla +\frac{j}{I}\frac{\partial }{\partial \psi })\,(\frac{1}{3}m{c}^{2}-p/n)\rangle ,\\ {Z}^{({\bf{Q}})}=\langle f(\frac{D}{Dt}+{\bf{c}}\cdot \nabla +\frac{j}{I}\frac{\partial }{\partial \psi })\,(\frac{1}{2}m{c}^{2}+{H}_{rot}-m\hat{h})\,{\bf{c}}\rangle .\end{array}$$

With **c** and *m* denoting the peculiar velocity of the molecule and the molecule mass, respectively. Here an abbreviation is used for the integration over **v** space with angular brackets $\langle \cdots \rangle =\int \,{\rm{d}}{\bf{v}}\cdots$. The symbol []^(2)^ stands for a traceless symmetric part of the tensor. To date, no approximations have been made in deriving the constitutive equations. Physically motivated conditions are imposed on the closure in the present work: $\nabla \cdot {{\boldsymbol{\Psi }}}^{()}$ are the high-order terms and changes faster than the evaluation of stress and heat flux. When such physically motivated closure is applied,

4 $$\nabla \cdot [\begin{array}{l}{{\boldsymbol{\Psi }}}^{({\boldsymbol{\Pi }})}\\ {{\boldsymbol{\Psi }}}^{({\rm{\Delta }})}\\ {{\boldsymbol{\Psi }}}^{({\bf{Q}})}\end{array}]=[\begin{array}{l}0\\ 0\\ 0\end{array}].$$

Note that this closure is different from that in previous studies^12,13^, in which both the unsteady term and convective term are removed. Then, the governing equation is reduced to

5 $$\begin{array}{l}\rho \frac{D}{Dt}\,\,[\begin{array}{l}{[1,\rho {\bf{u}},\rho E]}^{T}/\rho \\ {\boldsymbol{\Pi }}/\rho \\ ({\boldsymbol{\Pi }}+{\rm{\Delta }}{\bf{I}})/\rho \\ {\bf{Q}}/\rho \end{array}]+\nabla \cdot [\begin{array}{l}{[{\bf{u}},p{\bf{I}}+{\boldsymbol{\Pi }}+{\rm{\Delta }}{\bf{I}},(p{\bf{I}}+{\boldsymbol{\Pi }}+{\rm{\Delta }}{\bf{I}})\cdot {\bf{u}}+{\bf{Q}}]}^{T}\\ 0\\ 0\\ 0\end{array}]\\ \quad =\,[\begin{array}{l}{[0,\mathrm{0,}\mathrm{0]}}^{T}\\ {Z}^{({\boldsymbol{\Pi }})}-\frac{{\boldsymbol{\Pi }}}{\eta /p}q(\kappa )\\ {Z}^{({\rm{\Delta }})}-\frac{{\rm{\Delta }}{\bf{I}}}{{\eta }_{b}/p}q(\kappa )\\ {Z}^{({\bf{Q}})}-\frac{{\bf{Q}}}{\lambda /p{C}_{p}}q(\kappa )\end{array}]\end{array}$$

where the kinematic terms are defined as

6 $$\begin{array}{l}{Z}^{({\boldsymbol{\Pi }})}=\frac{{{\boldsymbol{\Pi }}}_{0}}{\eta /p}-\mathrm{2[}{\boldsymbol{\Pi }}\cdot \nabla {\bf{u}}{]}^{\mathrm{(2)}},\\ {Z}^{({\rm{\Delta }})}=\frac{3}{2\gamma ^{\prime} }\frac{{{\rm{\Delta }}}_{0}}{{\eta }_{b}/p}-2\gamma ^{\prime} ({\boldsymbol{\Pi }}+{\rm{\Delta }}{\bf{I}}):\nabla {\bf{u}}\\ {Z}^{({\bf{Q}})}=(1+\frac{{\boldsymbol{\Pi }}}{p})\cdot \frac{{{\bf{Q}}}_{0}}{\lambda /p{C}_{p}}+\frac{1}{Pr}\frac{{\bf{Q}}}{\lambda /p{C}_{p}}\cdot \frac{(-\eta \nabla {\bf{u}})}{p}\end{array}$$

The factor *q*(*κ*) appearing in the dissipative terms, which plays a critical role in the shock structure problem, is defined as

7 $$q(\kappa )\equiv \frac{\sinh \,\kappa }{\kappa },\,\kappa =\frac{{(m{k}_{B}T)}^{\mathrm{1/4}}}{\sqrt{2}pd}\,{[\frac{{\boldsymbol{\Pi }}:{\boldsymbol{\Pi }}}{2\eta }+\gamma ^{\prime} \frac{{{\rm{\Delta }}}^{2}}{{\eta }_{b}}+\frac{{\bf{Q}}\cdot {\bf{Q}}}{\lambda T}]}^{\mathrm{1/2}}.$$

In this expression, *η*, *η*_*b*_ and *λ* are the Chapman–Enskog shear viscosity, bulk viscosity, and thermal conductivity, respectively; *κ* is the first-order cumulant of the cumulant approximation for dissipation terms. *d* and *k*_*B*_ denote the diameter of the molecule and the Boltzmann constant, respectively. *C*_*p*_ denotes the heat capacity at constant pressure. *Pr* is the Prandtl number, and *γ*′ = (5 − 3*γ*)/2, where *γ* is the specific heat ratio. **Π**_0_,Δ_0_ and **Q**_0_ are determined by Newton’s law of shear and bulk viscosity and Fourier’s law of heat conduction.

8 $${{\boldsymbol{\Pi }}}_{0}=-2\eta {[{\bf{u}}]}^{\mathrm{(2)}},\,{{\rm{\Delta }}}_{0}=-{\eta }_{b}\nabla {\bf{u}},\,{{\bf{Q}}}_{0}=-\lambda \nabla T$$

Then, generalized hydrodynamic equations can be rewritten as follows,

9 $$\begin{array}{c}\frac{\partial {\bf{U}}}{\partial t}+\nabla \cdot {{\bf{F}}}_{{\rm{inv}}}({\bf{U}})+\nabla \cdot {{\bf{F}}}_{{\rm{vis}}}({\bf{U}},{\boldsymbol{\Pi }},{\rm{\Delta }},{\bf{Q}})=0\\ {\bf{U}}=(\begin{array}{c}\rho \\ \rho {\bf{u}}\\ \rho E\end{array}),{{\bf{F}}}_{{\rm{inv}}}({\bf{U}})=(\begin{array}{c}\rho {\bf{u}}\\ \rho {\bf{uu}}+p{\bf{I}}\\ (\rho E+p){\bf{u}}\end{array}),{{\bf{F}}}_{{\rm{vis}}}({\bf{U}},{\boldsymbol{\Pi }},{\rm{\Delta }},{\bf{Q}})=(\begin{array}{c}0\\ {\boldsymbol{\Pi }}+{\rm{\Delta }}{\bf{I}}\\ ({\boldsymbol{\Pi }}+{\rm{\Delta }}{\bf{I}})\cdot {\bf{u}}+{\bf{Q}}\end{array})\end{array}$$

10 $$\begin{array}{c}\frac{\partial {\boldsymbol{\Pi }}}{\partial t}+\nabla \cdot ({\boldsymbol{\Pi }}{\bf{u}})+\mathrm{2(}p+{\rm{\Delta }})\,{[\nabla {\bf{u}}]}^{\mathrm{(2)}}+\mathrm{2[}{\boldsymbol{\Pi }}\cdot \nabla {\bf{u}}{]}^{\mathrm{(2)}}+\frac{p}{\eta }{\boldsymbol{\Pi }}q(\kappa )=0\\ \frac{\partial ({\boldsymbol{\Pi }}+{\rm{\Delta }}{\bf{I}})}{\partial t}+\nabla \cdot (({\boldsymbol{\Pi }}+{\rm{\Delta }}{\bf{I}}){\bf{u}})+2\gamma ^{\prime} ({\boldsymbol{\Pi }}+{\rm{\Delta }}{\bf{I}}):\nabla {\bf{u}}+\frac{2}{3}\gamma ^{\prime} p\nabla \cdot {\bf{u}}+\frac{2}{3}\gamma ^{\prime} \frac{p}{{\eta }_{b}}{\rm{\Delta }}q(\kappa )=0\\ \frac{\partial {\bf{Q}}}{\partial t}+\nabla \cdot ({\bf{Qu}})+(p+{\rm{\Delta }}){C}_{p}T\nabla lnT+{\boldsymbol{\Pi }}\cdot {C}_{p}\nabla T+{\bf{Q}}\cdot \nabla {\bf{u}}+\frac{p{C}_{p}}{\lambda }{\bf{Q}}q(\kappa )=0\end{array}$$

Here, **I** is the unit second-rank tensor, and [**A**]^(2)^ is the traceless symmetric part of the second-rank tensor **A**.

11 $${[{\bf{A}}]}^{\mathrm{(2)}}=\frac{1}{2}({\bf{A}}+{{\bf{A}}}^{t})-\frac{1}{3}{\bf{I}}tr{\bf{A}}$$

If we use similar non-dimensional variables to those in previous work^10,12,13^, the dimensionless evolution equations in the generalized hydrodynamics equations can be written as,

12 $$\begin{array}{c}\frac{\partial {\bf{U}}}{\partial t}+\nabla \cdot {{\bf{F}}}_{{\rm{inv}}}({\bf{U}})+\nabla \cdot {{\bf{F}}}_{{\rm{vis}}}({\bf{U}},{\boldsymbol{\Pi }},{\rm{\Delta }},{\bf{Q}})=0\\ {\bf{U}}=(\begin{array}{c}\rho \\ \rho {\bf{u}}\\ \rho E\end{array}),{{\bf{F}}}_{{\rm{inv}}}({\bf{U}})=(\begin{array}{c}\rho {\bf{u}}\\ \rho {\bf{uu}}+\tfrac{p}{\gamma M{a}^{2}}{\bf{I}}\\ (\rho E+\tfrac{p}{\gamma M{a}^{2}}){\bf{u}}\end{array}),{{\bf{F}}}_{{\rm{vis}}}({\bf{U}},{\boldsymbol{\Pi }},{\rm{\Delta }},{\bf{Q}})=\tfrac{1}{Re}(\begin{array}{c}0\\ {\boldsymbol{\Pi }}+{\rm{\Delta }}{\bf{I}}\\ ({\boldsymbol{\Pi }}+{\rm{\Delta }}{\bf{I}})\cdot {\bf{u}}+\tfrac{1}{(\gamma -1)M{a}^{2}Pr}{\bf{Q}}\end{array})\end{array}$$

13 $$\begin{array}{c}\tfrac{\partial \hat{{\boldsymbol{\Pi }}}}{\partial t}+\nabla \cdot (\hat{{\boldsymbol{\Pi }}}{\hat{{\boldsymbol{\Pi }}}}_{0})+\mathrm{2(1}+{f}_{b}\hat{{\rm{\Delta }}}){\hat{{\boldsymbol{\Pi }}}}_{0}+\mathrm{2[}\hat{{\boldsymbol{\Pi }}}\cdot {\hat{{\boldsymbol{\Pi }}}}_{0}{]}^{\mathrm{(2)}}-2\hat{{\boldsymbol{\Pi }}}q(c\hat{R})=0\\ \tfrac{\partial (\hat{{\boldsymbol{\Pi }}}+{f}_{b}\hat{{\rm{\Delta }}}{\bf{I}})}{\partial t}+\nabla \cdot ((\hat{{\boldsymbol{\Pi }}}+{f}_{b}\hat{{\rm{\Delta }}}{\bf{I}}){\hat{{\boldsymbol{\Pi }}}}_{0})+2\gamma ^{\prime} (\hat{{\boldsymbol{\Pi }}}+{f}_{b}\hat{{\rm{\Delta }}}{\bf{I}}):{\hat{{\boldsymbol{\Pi }}}}_{0}+\tfrac{4\gamma ^{\prime} }{3{f}_{b}}{\hat{{\rm{\Delta }}}}_{0}+\tfrac{4\gamma ^{\prime} }{3{f}_{b}}\hat{{\rm{\Delta }}}q(c\hat{R})=0\\ \tfrac{\partial \hat{{\bf{Q}}}}{\partial t}+\nabla \cdot (\hat{{\bf{Q}}}{\hat{{\boldsymbol{\Pi }}}}_{0})+2Pr\mathrm{(1}+{f}_{b}\hat{{\rm{\Delta }}})\,{\hat{{\bf{Q}}}}_{0}+2Pr{\hat{{\bf{Q}}}}_{0}\cdot \hat{{\boldsymbol{\Pi }}}+\hat{{\bf{Q}}}\cdot {\hat{{\boldsymbol{\Pi }}}}_{0}-2Pr\hat{{\bf{Q}}}q(c\hat{R})=0\end{array}$$

Here,

14 $$\hat{{\boldsymbol{\Pi }}}=\frac{{N}_{\delta }}{p}{\boldsymbol{\Pi }},\,\hat{{\rm{\Delta }}}=\frac{{N}_{\delta }}{p}{\rm{\Delta }},\hat{{\bf{Q}}}=\frac{{N}_{\delta }}{p}\frac{{\bf{Q}}}{\sqrt{T[Pr(\gamma -\mathrm{1)}M{a}^{2}]/2}}$$

15 $${N}_{\delta }=\gamma \frac{M{a}^{2}}{Re}$$

16 $${\hat{R}}^{2}=\hat{{\boldsymbol{\Pi }}}:\hat{{\boldsymbol{\Pi }}}+2{f}_{b}\gamma ^{\prime} \hat{{\rm{\Delta }}}+\hat{{\bf{Q}}}\cdot \hat{{\bf{Q}}}$$

*Ma* and *Re* are dimensionless gas dynamic parameters: Mach and Reynolds numbers, respectively. The constant *c*, which is given by the molecular model, has a value of 1.0138 for the Maxwell model and 1.0179 for the hard sphere model.

By introducing the auxiliary variables **S** in vector form, the dimensionless generalized hydrodynamics can be rewritten as

17 $$\{\begin{array}{l}\frac{\partial {\bf{U}}}{\partial t}+\nabla \cdot {{\bf{F}}}_{{\rm{inv}}}({\bf{U}})+\nabla \cdot {{\bf{F}}}_{{\rm{vis}}}({\bf{U}},{\bf{S}})=0\\ \frac{\partial {\bf{S}}}{\partial t}+\nabla \cdot {\bf{G}}({\bf{S}})+{\bf{H}}({\bf{S}})=0\end{array}$$

Here,

18 $${\bf{U}}=(\begin{array}{c}\rho \\ \rho {\bf{u}}\\ \rho E\end{array}),{{\bf{F}}}_{{\rm{inv}}}=(\begin{array}{c}\rho {\bf{u}}\\ \rho {\bf{uu}}+\frac{p}{\gamma M{a}^{2}}{\bf{I}}\\ (\rho E+\frac{p}{\gamma M{a}^{2}})\,{\bf{u}}\end{array}),{{\bf{F}}}_{{\rm{vis}}}=\frac{1}{Re}\,(\begin{array}{c}0\\ {\boldsymbol{\Pi }}+{\rm{\Delta }}{\bf{I}}\\ ({\boldsymbol{\Pi }}+{\rm{\Delta }}{\bf{I}})\cdot {\bf{u}}+\frac{1}{(\gamma -\mathrm{1)}M{a}^{2}Pr}{\bf{Q}}\end{array})$$

19 $$\begin{array}{c}{\bf{S}}=(\begin{array}{c}\hat{{\boldsymbol{\Pi }}}\\ \hat{{\boldsymbol{\Pi }}}+{f}_{b}\hat{{\rm{\Delta }}}{\bf{I}}\\ \hat{{\bf{Q}}}\end{array}),{\bf{G}}({\bf{S}})=(\begin{array}{c}\hat{{\boldsymbol{\Pi }}}{\hat{{\boldsymbol{\Pi }}}}_{0}\\ (\hat{{\boldsymbol{\Pi }}}+{f}_{b}\hat{{\rm{\Delta }}}{\bf{I}}){\hat{{\boldsymbol{\Pi }}}}_{0}\\ \hat{{\bf{Q}}}{\hat{{\boldsymbol{\Pi }}}}_{0}\end{array}),\\ {\bf{H}}({\bf{S}})=(\begin{array}{c}\mathrm{2(1}+{f}_{b}\hat{{\rm{\Delta }}}){\hat{{\boldsymbol{\Pi }}}}_{0}+\mathrm{2[}\hat{{\boldsymbol{\Pi }}}\cdot {\hat{{\boldsymbol{\Pi }}}}_{0}{]}^{\mathrm{(2)}}-2\hat{{\boldsymbol{\Pi }}}q(c\hat{R})\\ 2\gamma ^{\prime} (\hat{{\boldsymbol{\Pi }}}+{f}_{b}\hat{{\rm{\Delta }}}{\bf{I}}):{\hat{{\boldsymbol{\Pi }}}}_{0}+\frac{4\gamma ^{\prime} }{3{f}_{b}}{\hat{{\rm{\Delta }}}}_{0}+\frac{4\gamma ^{\prime} }{3{f}_{b}}\hat{{\rm{\Delta }}}q(c\hat{R})\\ 2Pr\mathrm{(1}+{f}_{b}\hat{{\rm{\Delta }}})\,{\hat{{\bf{Q}}}}_{0}+2Pr{\hat{{\bf{Q}}}}_{0}\cdot \hat{{\boldsymbol{\Pi }}}+\hat{{\bf{Q}}}\cdot {\hat{{\boldsymbol{\Pi }}}}_{0}-2Pr\hat{{\bf{Q}}}q(c\hat{R})\end{array})\end{array}$$

The remainder of this study solves Eqs (17–19) within the discontinuous Galerkin (DG) framework. To differentiate from the previous work^12,13^, we name Eqs (17–19) the Nonlinear Coupled Constitutive Model (NCCM).

## Numerical Algorithms of the Nonlinear Coupled Constitutive Model

Although the NCCM as shown in Eqs (9 and 10) is envisioned to solve all the regimes within a unified framework to treat high and low Knudsen number flows, these equations are quiet high nonlinear; therefore an appropriate numerical algorithm is required. Unfortunately, the Finite Volume Method (FVM) is limited to second-order accuracy at best, and in particular, it suffers a noticeable degradation in low Mach number flows. This deficiency is critical and sometimes it overcomes the attractive feature of highly coupled nonlinear equations in constitutive relations of Eq. (10).

It is fortunate that the DG method has been popular recently as a numerical technique for solving hyperbolic conservation laws^14,15^. It provides an alternative and attractive way to solve generalized hydrodynamic equations. DG obtains high accuracy by using a higher-order polynomial approximation within an element. This substantially differs from FVM and Finite Difference Methods (FDM) in which high accuracy is achieved by using wide stencils^16^. This is the primary reason that we employ the DG method in the present study. Furthermore, the DG method may be divided into modal^17–19^ or nodal^20–22^, depending on the basis function used in the scheme. The degree of freedom in nodal DG corresponds to the solution at a coordinate within the element because the shape functions are of the nodal form: for example, Lagrange interpolation. The primary advantage of modal DG is that the basis numbers are not tied to the geometry of an element since the degrees of freedom are the modal shape functions^23^. Cockburn and Shu have contributed much to the construction of the framework for DG based on the modal approach^14,24,25^. It has been successfully extended to apply to a variety of multidimensional problems, such as fluid dynamics, acoustics^26^, and magneto-hydrodynamics^27^. Therefore, the modal DG method is employed in this study.

A mixed formulation is proposed by Bassi^17^ *et al*. for the treatment of the second-order viscous terms to solve the Navier-Stokes equations, which differs from the conventional DG method^28^. The formulation introduces auxiliary variables to resolve the governing equations as a first-order coupled system for the local DG approach. In this work, we extend mixed DG to solve the Nonlinear Coupled Constitutive Model. The coupled systems for Nonlinear Coupled Constitutive Model are,

20 $$\{\begin{array}{l}\frac{\partial {\bf{U}}}{\partial t}+\nabla \cdot {{\bf{F}}}_{{\rm{inv}}}({\bf{U}})+\nabla \cdot {{\bf{F}}}_{{\rm{vis}}}({\bf{U}},{\bf{S}})=0\\ \frac{\partial {\bf{S}}}{\partial t}+\nabla \cdot {\bf{G}}({\bf{S}})+{\bf{H}}({\bf{S}})=0\end{array}$$

The numerical solutions of **U** and **S** are approximated by **U**_*h*_ and **S**_*h*_ in the local element Ω,

$${{\bf{U}}}_{h}({\bf{x}},t)=\sum _{i=1}^{K}\,{U}^{i}(t)\,{\phi }_{i}({\bf{x}}),{{\bf{S}}}_{h}({\bf{x}},t)=\sum _{i=1}^{K}\,{S}^{i}(t)\,{\phi }_{i}({\bf{x}})$$

where *φ*_*i*_(**x**) is the basis function. The number of basis *K* depends on the order of approximation *I* (*P* ^*I*^ representing the different order of approximations such as *P* ^1^ and *P* ^2^). The relation between *K* and *I* is given by

$$K=\frac{(I+1)\,(I+2)}{2}\quad (in\,2D)\quad K=\frac{(I+1)\,(I+2)\,(I+3)}{6}\quad (in\,3D)$$

The basis functions are defined in a global sense, meaning that the same basis functions are used in each local element. The coupled system (20) is multiplied by the basis function ***φ*** and then integrated by parts in derivative terms over element Ω, and then the weak formulation of the coupled system can be derived to find **U**_*h*_ and **S**_*h*_

21 $$\{\begin{array}{l}\frac{\partial }{\partial t}\,{\int }_{{\rm{\Omega }}}\,{{\bf{U}}}_{h}{\boldsymbol{\phi }}{\rm{d}}V-{\int }_{{\rm{\Omega }}}\,\nabla {\boldsymbol{\phi }}{{\bf{F}}}_{{\rm{inv}}}{\rm{d}}V+{\int }_{{\rm{\Gamma }}}\,{\boldsymbol{\phi }}{{\bf{F}}}_{{\rm{inv}}}{\rm{d}}s-{\int }_{{\rm{\Omega }}}\,\nabla {\boldsymbol{\phi }}{{\bf{F}}}_{{\rm{vis}}}{\rm{d}}V+{\int }_{{\rm{\Gamma }}}\,{\boldsymbol{\phi }}{{\bf{F}}}_{{\rm{vis}}}{\rm{d}}s=0,\\ \frac{\partial }{\partial t}\,{\int }_{{\rm{\Omega }}}\,{{\bf{S}}}_{h}{\boldsymbol{\phi }}{\rm{d}}V+{\int }_{{\rm{\Omega }}}\,[\nabla {\boldsymbol{\phi }}{\bf{G}}({{\bf{S}}}_{h})+{\boldsymbol{\phi }}{\bf{H}}({{\bf{S}}}_{h})]\,{\rm{d}}V-{\int }_{{\rm{\Gamma }}}\,{\boldsymbol{\phi }}{\bf{G}}({{\bf{S}}}_{h})\,{\rm{d}}s=0.\end{array}$$

where Γ denotes the boundaries of the element Ω.

The equations for **S** are solved first to compute the nonconservative variables such as viscous stress, excess normal stress and heat flux, where the variable **U**(**x**, *t*) is updated at each time step. The boundary integrals of each element are replaced by a numerical flux function as follows. For inviscid terms in equations for **U**(**x**, *t*), the local Lax-Friedrichs (LxF) flux^12,13^, **h**_inv_, is applied.

22 $${{\bf{h}}}_{{\rm{inv}}}({{\bf{U}}}^{-},{{\bf{U}}}^{+},{\bf{n}})=\frac{1}{2}[{{\bf{F}}}_{{\rm{inv}}}({{\bf{U}}}^{-})+{{\bf{F}}}_{{\rm{inv}}}({{\bf{U}}}^{+})-{C}_{{\rm{inv}}}({{\bf{U}}}^{+}-{{\bf{U}}}^{-})]$$

where

$${C}_{{\rm{inv}}}=max(|{u}^{-}|+\frac{{c}_{s}^{-}}{Ma},|{u}^{+}|+\frac{{c}_{s}^{+}}{Ma})$$

and *c*_*s*_ = *T* ^1/2^ is the speed of sound at the element interface. The signs − and + denote the insides and outsides of an elemental interface. For the viscous term, the positivity-preserving flux proposed by Zhang^29,30^, **h**_vis_, is applied.

23 $${{\bf{h}}}_{{\rm{vis}}}({{\bf{U}}}^{-},{{\bf{S}}}^{-},{{\bf{U}}}^{+},{{\bf{S}}}^{+};{\bf{n}})\cong {\int }_{{\rm{\Gamma }}}\,{\boldsymbol{\phi }}{{\bf{F}}}_{{\rm{vis}}}{\rm{d}}x=\frac{1}{2}[{{\bf{F}}}_{{\rm{vis}}}({{\bf{U}}}^{-},{{\bf{S}}}^{-})+{{\bf{F}}}_{{\rm{vis}}}({{\bf{U}}}^{+},{{\bf{S}}}^{+})-{C}_{{\rm{vis}}}({{\bf{U}}}^{+}-{{\bf{U}}}^{-})]$$

where

$$\begin{array}{rcl}{C}_{{\rm{vis}}} & = & max({\beta }^{-},{\beta }^{+}),\\ \,\,\,\beta  & = & max\tfrac{1}{2\rho eRe(\gamma -\mathrm{1)}M{a}^{2}Pr}(\sqrt{{|{\bf{Q}}\cdot {\bf{n}}|}^{2}+(\gamma -\mathrm{1)}M{a}^{2}Pr2e{\Vert {\boldsymbol{\Pi }}\cdot {\bf{n}}\Vert }^{2}}+|{\bf{Q}}\cdot {\bf{n}}|)\\ \,\rho e & = & \tfrac{p}{\gamma (\gamma -\mathrm{1)}M{a}^{2}}\end{array}$$

The central flux is applied for the terms in the equations for **S**,

24 $${{\bf{h}}}_{G,H}({{\bf{S}}}^{-},{{\bf{S}}}^{+};{\bf{n}})\cong {\int }_{{\rm{\Gamma }}}\,{\boldsymbol{\phi }}{\bf{G}}({{\bf{S}}}_{h}){\rm{d}}s=\frac{1}{2}[{{\bf{G}}}^{-}+{{\bf{G}}}^{+}]$$

The volume integrals within the element Ω are resolved by the Gaussian quadrature.

Finally, the coupled system (20) can be written in semi-discrete form as

25 $$\begin{array}{l}{\bf{L}}\frac{\partial {\bf{S}}}{\partial t}={{\bf{R}}}_{S}({\bf{S}}),\\ {\bf{L}}\frac{\partial {\bf{U}}}{\partial t}={{\bf{R}}}_{U}({\bf{U}},{\bf{S}}\mathrm{).}\end{array}$$

Which can be solved by the multi-order Runge-Kutta time integration. Because of the orthogonality property of basis functions, the diagonal matrix **L** is readily invertible.

In the initial condition, the coefficients of conserved variables are specified on the basis of farfiled,

$${U}^{1}(t=\mathrm{0)}={{\bf{U}}}_{farfield},{U}^{i}(t=\mathrm{0)}=\mathrm{0(}i=2,\ldots ,K).$$

The coefficients of **S** are set to zero;

$${S}^{i}(t=\mathrm{0)}=\mathrm{0(}i=1,\ldots ,K\mathrm{).}$$

The time step Δ*t* is computed^18^ as

26 $${\rm{\Delta }}t=min({\rm{\Delta }}{t}_{i}),{\rm{\Delta }}{t}_{i}=\frac{1}{{(I+\mathrm{1)}}^{2}}\frac{{\rm{\Delta }}{\bf{x}}CFL}{|u|+\frac{a}{Ma}+\frac{\eta }{\rho {\rm{\Delta }}{\bf{x}}Re}}$$

where *CFL* is the Courant-Friedrichs-Lewy condition (*CFL* ≤ 1).

In summary, the DG scheme for the Nonlinear Coupled Constitutive Model is conducted with the following steps:

1. Compute ${\hat{{\boldsymbol{\Pi }}}}_{0}$, ${\hat{{\rm{\Delta }}}}_{0}$ and ${\hat{{\bf{Q}}}}_{0}$ based on the approximation of conserved variables **U**.
2. Compute **Π**, Δ and **Q** based on the approximation of nonconserved variables **S**.
3. Compute the flux and integration of the evolution equations for **S** by the Gaussian quadrature. Update the approximation of **S** by the Runge-Kutta method.
4. According to the updated approximation of **S**, update **Π**, Δ and **Q** in the evolution equations for **U**. Update the approximation of **U** by the Runge-Kutta method.
5. Return to step (1) until the convergent error is satisfied.

## A. Basis Functions

The Dubiner basis functions are derived from the Jacobi polynomial in the interval *x* ∈ [−1, 1]^31^:

27 $${P}_{n}^{\alpha ,\beta }\,(x)=\frac{{(-\mathrm{1)}}^{n}}{{2}^{n}n!}{\mathrm{(1}-x)}^{-\alpha }\,{\mathrm{(1}+x)}^{-\beta }\,\frac{{{\rm{d}}}^{n}}{{\rm{d}}{x}^{n}}[{(1-x)}^{n+\alpha }\,{(1+x)}^{n+\beta }]$$

It can be constructed as products of up to three original functions,

$$\begin{array}{rcl}\,{{\rm{\Theta }}}_{i}^{a}(x) & = & {P}_{i}^{\mathrm{0,0}}(x),\\ \,{{\rm{\Theta }}}_{ij}^{b}(x) & = & {(\frac{1-x}{2})}^{i}{P}_{j}^{2i+\mathrm{1,0}}(x),\\ {{\rm{\Theta }}}_{ijk}^{c}(x) & = & {(\frac{1-x}{2})}^{i+j}{P}_{k}^{2i+2j+\mathrm{2,0}}(x\mathrm{).}\end{array}$$

The basis functions *φ*_(*i*,*j*)_(*r*, *s*) on the triangle element {(*r*, *s*), −1 ≤ *r*, *s*; *r* + *s* ≤ 0} are defined as the following products of the original function:

28 $${\phi }_{(i,j)}(r,s)={{\rm{\Theta }}}_{i}^{a}(e)\cdot {{\rm{\Theta }}}_{ij}^{b}(f)$$

where $e=\frac{1+2r+s}{1-s}$, *f* = *s*. The basis functions *φ*_(*i*,*j*,*k*)_(*r*, *s*, *t*) on the tetrahedron element {(*r*, *s*, *t*), 0 ≤ *r*, *s*, *t*; *r* + *s* + *t* ≤ 1} can be expressed as follows,

29 $${\phi }_{(i,j,k)}(r,s,t)={{\rm{\Theta }}}_{i}^{a}(e)\cdot {{\rm{\Theta }}}_{ij}^{b}(f)\cdot {{\rm{\Theta }}}_{ijk}^{c}(g)$$

Here, $$e=\frac{-1+2r+s+t}{1-s-t}$$, $$f=\frac{-1+2s+2t}{1-t}$$, *g* = −1 + 2*t*.

In two dimensions, the following standard triangle and quadrilateral are considered

30 $$T=\{(r,s),-1\le r,s;r+s\le 0\}$$

and

31 $$R=\{(a,b),-1\le a,b\le 1\}$$

**Figure 1:**
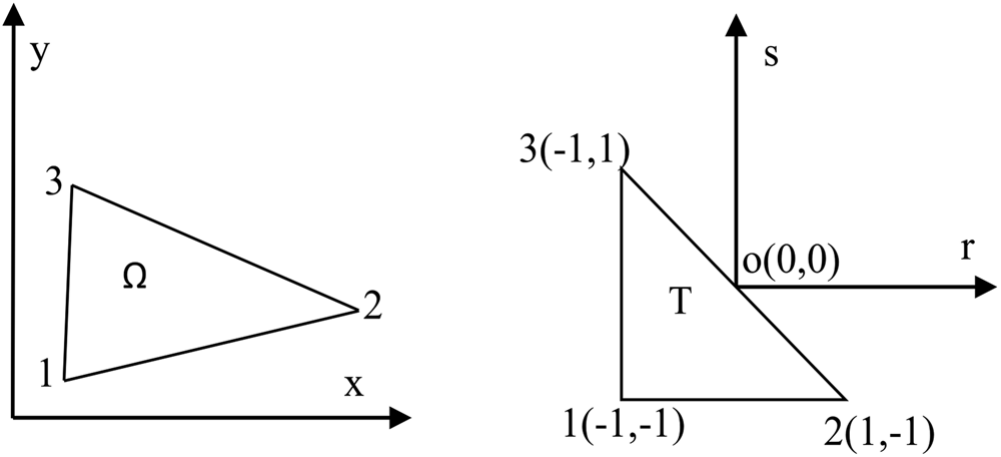
Transform between the arbitrary triangle Ω and the standard triangle T.

**Figure 2:**
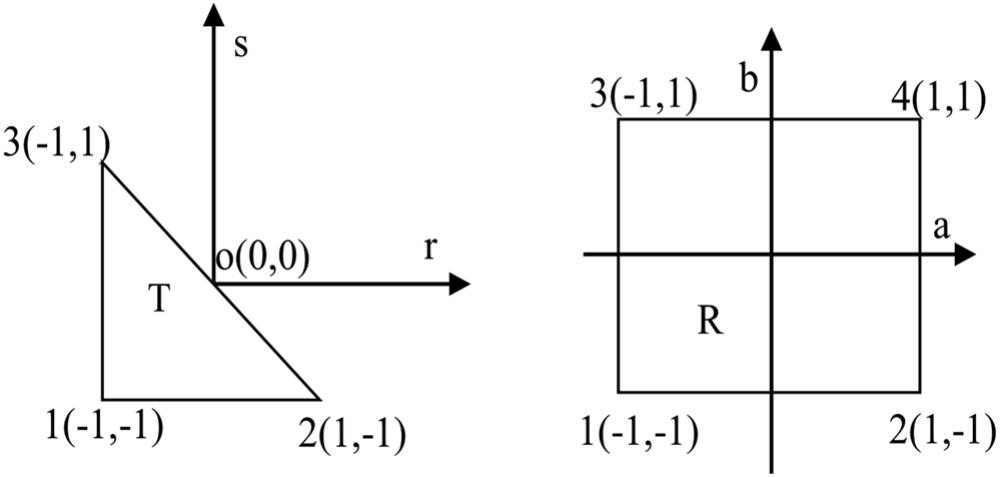
Transform between the standard triangle T and the standard quadrilateral R.

The basis functions can be equivalently written in *T*, *R* or the arbitrary triangle element Ω of the Cartesian coordinate system (*x*, *y*) because of the transforms (see Figs 1 and 2).

Ω → *T*:

32 $$(x,y)\to \{\begin{array}{l}r=\tfrac{2x({y}_{3}-{y}_{1})-2y({x}_{3}-{x}_{1})+({x}_{3}-{x}_{1})\,({y}_{3}+{y}_{2})-({y}_{3}-{y}_{1})\,({x}_{3}+{x}_{2})}{2A}\\ s=\tfrac{2x({y}_{2}-{y}_{1})-2y({x}_{2}-{x}_{1})+({x}_{2}-{x}_{1})\,({y}_{3}+{y}_{2})-({y}_{2}-{y}_{1})\,({x}_{3}+{x}_{2})}{-2A}\end{array}$$

*T* → *R*:

33 $$(r,s)\to \{\begin{array}{l}a=2\frac{1+r}{1-s}-1\\ b=s\end{array}$$

Here, *A* is the area of the arbitrary triangle Ω in the Cartesian coordinate system (*x*, *y*).

We consider the following standard tetrahedral in three dimensions,

34 $$T=\{(r,s,t\mathrm{),0}\le r,s,t;r+s+t\le 1\}$$

**Figure 3:**
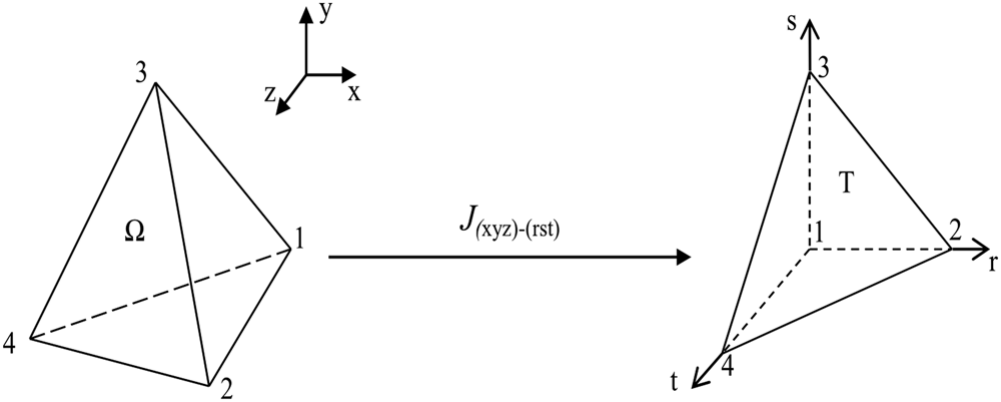
Transform between the arbitrary tetrahedral Ω and the standard tetrahedral T.

Similar to the case in two dimensions, the basis functions can also be equivalently written both in T and the arbitrary tetrahedral element Ω of the Cartesian coordinate system (*x*, *y*, *z*) due to the transforms (see Fig. 3).

Ω → *T*:

35 $$(x,y,z)\to \{\begin{array}{l}r=\frac{|\begin{array}{ccc}x-{x}_{1} & {x}_{3}-{x}_{1} & {x}_{4}-{x}_{1}\\ y-{y}_{1} & {y}_{3}-{y}_{1} & {y}_{4}-{y}_{1}\\ z-{z}_{1} & {z}_{3}-{z}_{1} & {z}_{4}-{z}_{1}\end{array}|}{6{V}_{e}}\\ s=\frac{|\begin{array}{ccc}{x}_{2}-{x}_{1} & x-{x}_{1} & {x}_{4}-{x}_{1}\\ {y}_{2}-{y}_{1} & y-{y}_{1} & {y}_{4}-{y}_{1}\\ {z}_{2}-{z}_{1} & z-{z}_{1} & {z}_{4}-{z}_{1}\end{array}|}{6{V}_{e}}\\ t=\frac{|\begin{array}{ccc}{x}_{2}-{x}_{1} & {x}_{3}-{x}_{1} & x-{x}_{1}\\ {y}_{2}-{y}_{1} & {y}_{3}-{y}_{1} & y-{y}_{1}\\ {z}_{2}-{z}_{1} & {z}_{3}-{z}_{1} & z-{z}_{1}\end{array}|}{6{V}_{e}}\end{array}$$

Here, *V*_*e*_ denotes the volume of the arbitrary tetrahedral Ω in the Cartesian coordinate system (*x*, *y*).

## B. Numerical integration

In the weak formulation of the governing equations (Eq. (21)), the volume and surface integrals must be approximated. Because the accuracy of the DG method is affected by the order of the numerical integration, it is expected that the choice of quadrature rule limits the order of the DG method. Therefore, choosing an appropriate numerical integration method is essential to obtain highly accurate DG solutions. In the present work, the Gaussian Legendre quadrature rule is employed to solve non-linear integrals inside the element, and over the element interfaces^32^. In two dimensions, we use 3 Gauss points for *P* ^1^ and 5 Gauss points for *P* ^2^ in the integration over the element interfaces and 9 for *P* ^1^ and 25 for *P* ^2^ in integrals inside the element. These numbers of Gauss points are 9, 25 and 11, 21 respectively in the integration of three dimensions.

## C. Limiter

The implementation of limiter is very important in the DG framework. In the present study, we extend Cockburn and Shu’s idea^14,24,25^ direction-by-direction both in two and three dimensions for *P* ^1^ approximation. A coupled slope limiter is employed in *P* ^2^ approximation.

## D. Slip boundary conditions

The Langmuir boundary^33,34^ is based on the Langmuir adsorption isotherm and considers the interfacial gas-surface. It gives good results for laminar and rarefied gas flows. A coverage fraction, *α*(0 ≤ *α* ≤ 1), of molecules reaching thermal equilibrium on the surface can be expressed, in dimensional form, as

36 $$\begin{array}{ll}\alpha =\frac{\beta p}{1+\beta p} & for\,monatomic\\ \alpha =\frac{\sqrt{\beta p}}{1+\sqrt{\beta p}} & for\,diatomic\end{array}$$

where *p* is the surface pressure and *β* depends on the surface temperature *T*_*w*_. By considering the gas-surface molecular interaction process as a chemical reaction, the parameter *β* can be expressed,

37 $$\beta =\sqrt{\frac{\pi }{32}}\frac{\pi }{{c}^{2}}\frac{{T}_{r}}{{T}_{w}}\,exp(\frac{{D}_{e}}{{R}_{u}{T}_{w}})\frac{1}{{p}_{r}Kn}$$

where *c* is gas constant, *p*_*r*_ and *T*_*r*_ are reference pressure and temperature, *R*_*u*_ is the universal gas constant, *R*_*u*_ = 8314 *J*/(*mol* · *K*), *D*_*e*_ is the heat of adsorption, *D*_*e*_ = 5255 *J*/*mol* for Ar-Al molecular interaction model and *K*_*n*_ is the global Knudsen number. The slip and jump Langmuir boundary conditions are determined according to the fraction, *α*,

38 $$\begin{array}{c}\,{\bf{u}}=\alpha {{\bf{u}}}_{w}+\mathrm{(1}-\alpha ){{\bf{u}}}_{g}\\ T=\alpha {T}_{w}+\mathrm{(1}-\alpha ){T}_{g}\end{array}$$

where **u** is the velocity vector, **u**_*w*_ is the wall velocity, and **u**_*g*_ and *T*_*g*_ are gas velocity and temperature at the reference location, respectively. All reference values are from the farfield conditions in the present work. As shown in Eq. (37), the jumping coefficient approaches 1 with the decrease in the Knudsen number (*K*_*n*_), and then it becomes a nonslip boundary condition.

Because of the properties of mixed DG, the wall boundary conditions can be imposed in a stable way. The inviscid fluxes in Eq. (22) are equal to the contribution of pressure, slip velocity and jump temperature. All the values are taken from the internal boundary state.

39 $${{{\bf{h}}}_{{\rm{inv}}}({{\bf{U}}}^{-},{{\bf{U}}}^{+};{\bf{n}})|}_{{\rm{wall}}}={{\bf{F}}}_{{\rm{inv}}}({{\bf{U}}}_{{\rm{wall}}})$$

The viscous flux in Eqs 23 and 24, are obtained by the value of the auxiliary unknown parameters **S**,

40 $${{{\bf{h}}}_{G,H}({{\bf{S}}}^{-},{{\bf{S}}}^{+};{\bf{n}})|}_{{\rm{wall}}}={{\bf{S}}}_{{\rm{wall}}}$$

41 $${{{\bf{h}}}_{{\rm{vis}}}({{\bf{U}}}^{-},{{\bf{S}}}^{-},{{\bf{U}}}^{+},{{\bf{S}}}^{+};{\bf{n}})|}_{{\rm{wall}}}={{\bf{F}}}_{{\rm{vis}}}({{\bf{U}}}_{{\rm{wall}}},{{\bf{S}}}_{{\rm{wall}}})$$

## Results

Verification and validation are the critical issues for computational models. In this section, we present several numerical results in two and three dimensions. The NCCM model is expected to develop a unified scheme for investigating both continuum and rarefied gas flows. It is accepted that NSF equations can be used only to solve the problem of continuum gas flows. A particle-based method DSMC, is widely used for in the investigation of rarefied effects. We compare the results obtained from the present DG-NCCM with those from DG-NSF, DSMC, and benchmark experiments in continuum and rarefied states.

We first compared the *P* ^1^ and *P* ^2^ results from the DG-NSF and DG-NCCM methods and found that both methods can capture the flow structures. Although the time resource for *P* ^2^ in DG is a little expensive, researchers are usually interested in the accuracy resulting from the high-order approximation. Therefore, all of the following studies are *P* ^2^ results even if there is a slight difference between the *P* ^1^ and *P* ^2^ results.

### A. The gas flow around a 2D cylinder

Flow over a cylinder was chosen as the cases study for the validation of the present method since a number of studies by others serve well for comparisons^35^. For all computations, the cylinder is placed in the middle of a 30*d* × 30*d* circle domain and *d* is the cylinder diameter. After a grid independence test, we place 100 nodes and 300 nodes in circumferential and radial directions, respectively. The outside cycle is chosen as the far-field boundary, and the wall is set to be the Langmuir boundary.

**Figure 4:**
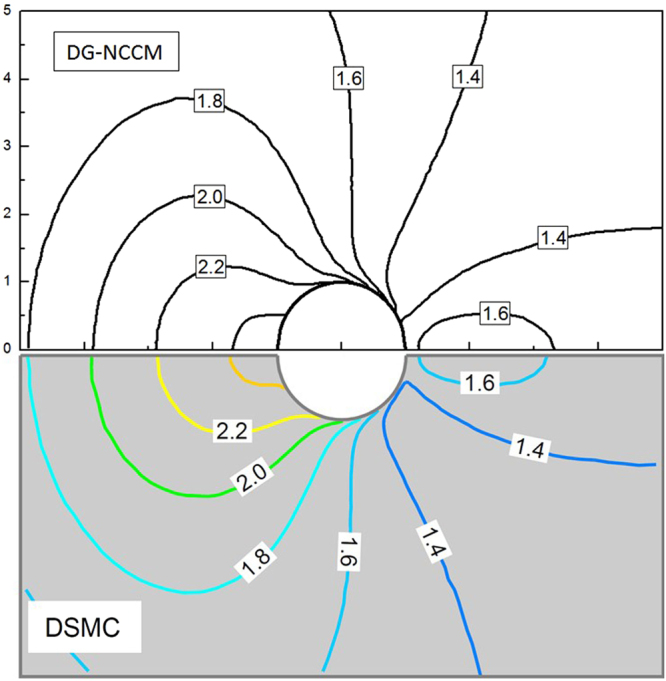
Temperature contours obtained with the present DG-NCCM and DSMC at Ma = 2.0 and Kn = 1.0.

In this study, supersonic flows around a cylinder at a free stream Mach number of 2.0 and Knudsen numbers from 0.01 to 1.0 are studied. The Knudsen number is estimated based on the diameter of the cylinder. The results from the present DG-NCCM, DG-NSF, and DSMC methods are compared. Figure 4 shows the comparison of temperature contours obtained with the DG-NCCM and DSMC at a high free stream Knudsen number of 1.0. Very good agreement between the DG-NCCM results and the DSMC results is observed.

**Figure 5:**
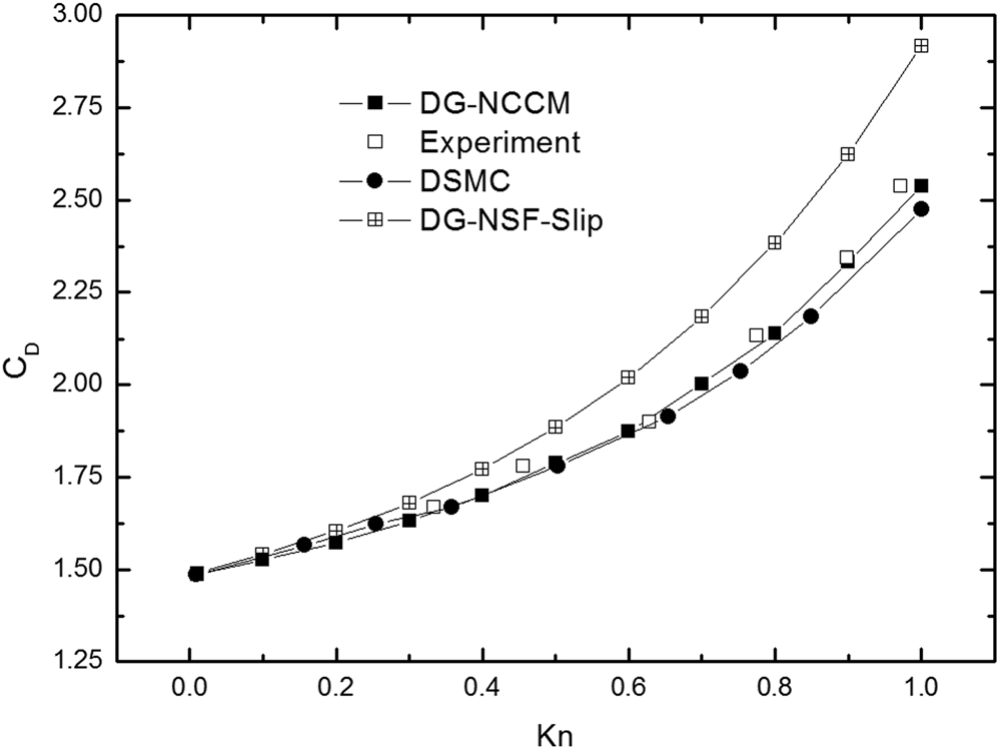
The drag coefficients of cylinder vs Knudsen numbers at Ma = 2.0.

Figure 5 shows the drag coefficients as a function of the Knudsen number. The results of experiments^36^ are also plotted for comparison. The results from DG-NSF agree well with the experiments at Knudsen numbers less than 0.1. However, this model over-predicts the drag coefficients for high Knudsen number cases. This result agrees with the conclusion of Hadjiconstantinou^5^ in that the results from NSF with slip boundary conditions are acceptable for Kn less than 0.1. When the Knudsen number becomes appreciable and greater than 0.1, the NSF fails to describe the gas flows. The agreement from DG-NCCM is expected to be observed with DSMC and with experiments over the whole studied region.

### B. The gas flow around a 3D sphere

The flow past a sphere at low Reynolds numbers has been extensively studied in experiments and by simulation. There are numerous benchmark cases to validate the new models for gas flows. Experimentally, it has been found that a recirculating zone (or vortex ring) develops close to the rear stagnation point at low Reynolds numbers. With a further increase in the Reynolds number, this recirculating zone or wake expands^36,37^. The drag coefficients of a sphere have also been experimentally studied in Ross and Willmarth’s work^38^ for Reynolds numbers up to 200. Therefore, we conducted the numerical studies of gas flows around a sphere to validate the capability of the present DG-NCCM scheme.

**Figure 6:**
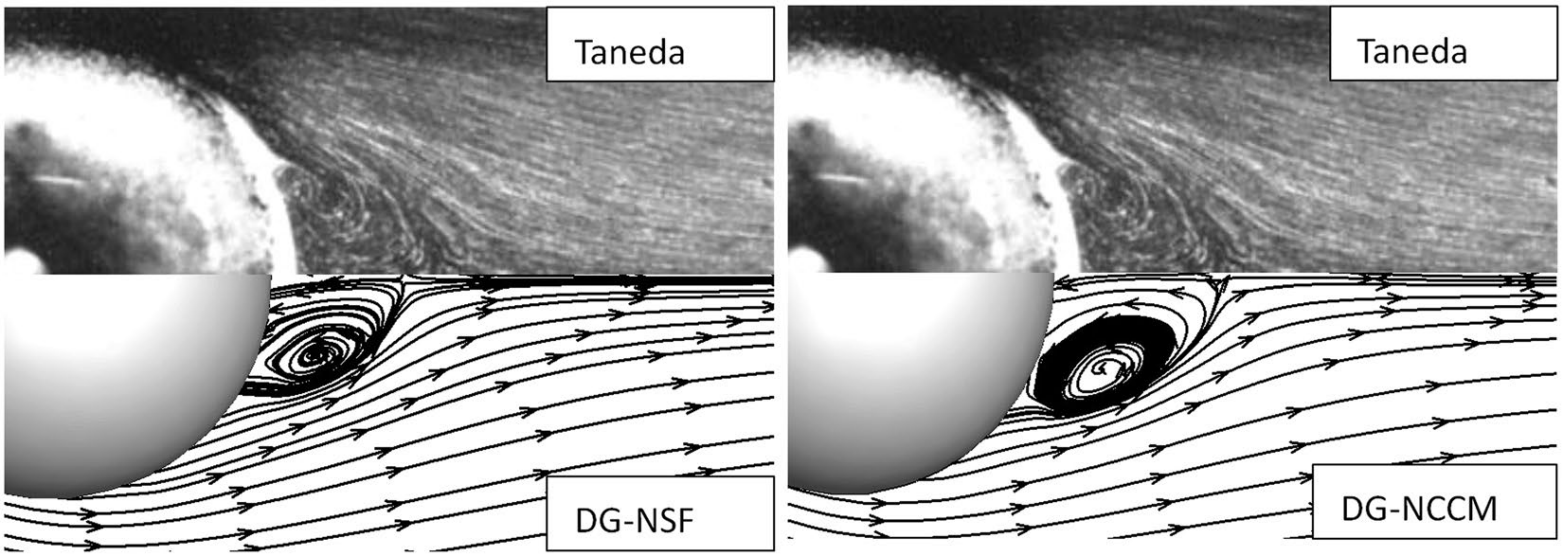
The stream lines of the gas flows around a sphere at Ma = 0.01, Re = 37.7.

**Figure 7:**
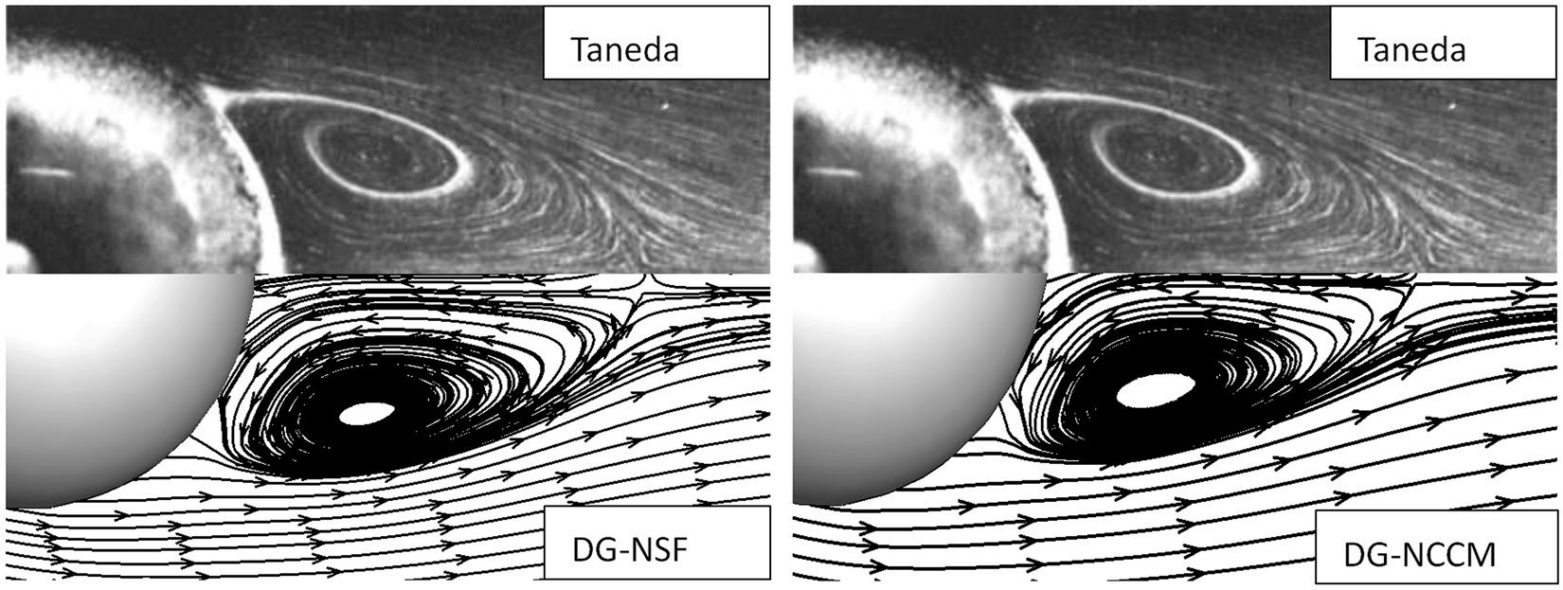
The stream lines of the gas flows around a sphere at Ma = 0.01, Re = 73.6.

**Figure 8:**
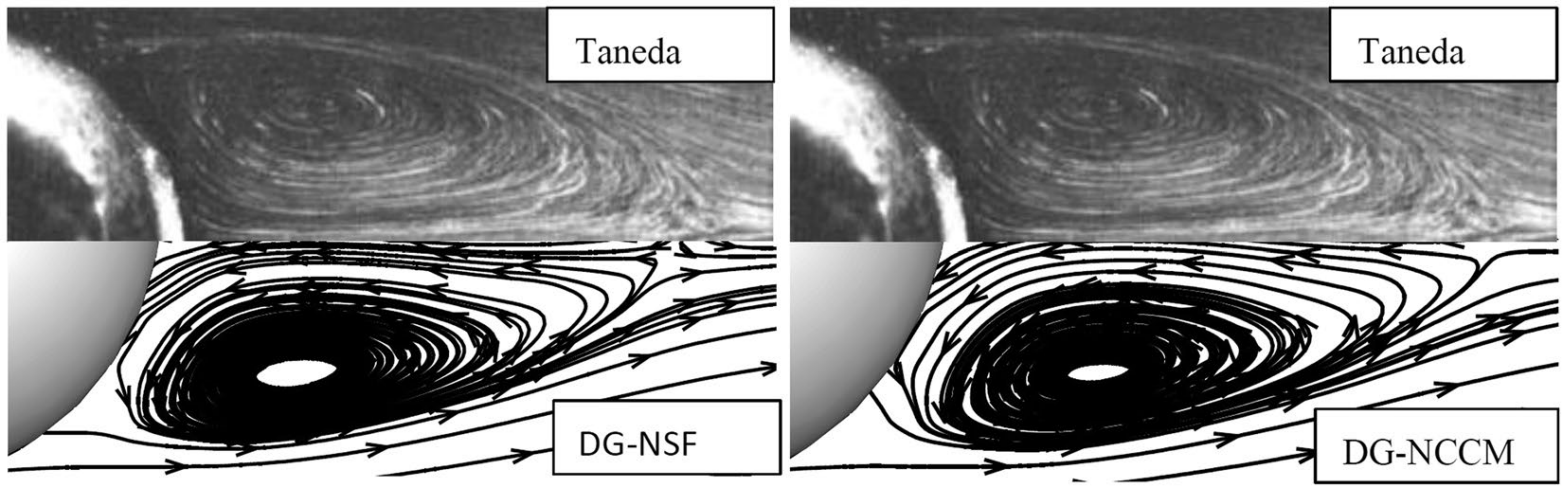
The stream lines of the gas flows around a sphere at Ma = 0.01, Re = 118.

In the present study, the sphere cylinder is placed in the middle of a 30*d* × 30*d* sphere calculation domain and *d* is the sphere diameter. After checking the grid independence, a set of unstructured meshes with 75 × 75 cells in wall-conforming direction and 150 cells in wall-normal direction, is implemented in the computational domain. Figures 6, 7 and 8 illustrate the stream lines of the gas flows around a sphere at the Reynolds numbers of *Re* = 37.7, 73.6, and 118 in *Ma* = 0.01. The comparisons show that the present DG-NCCM can capture the vortex structure and has good agreement with the result of the experimental study^39^ for the cases studied.

**Figure 9:**
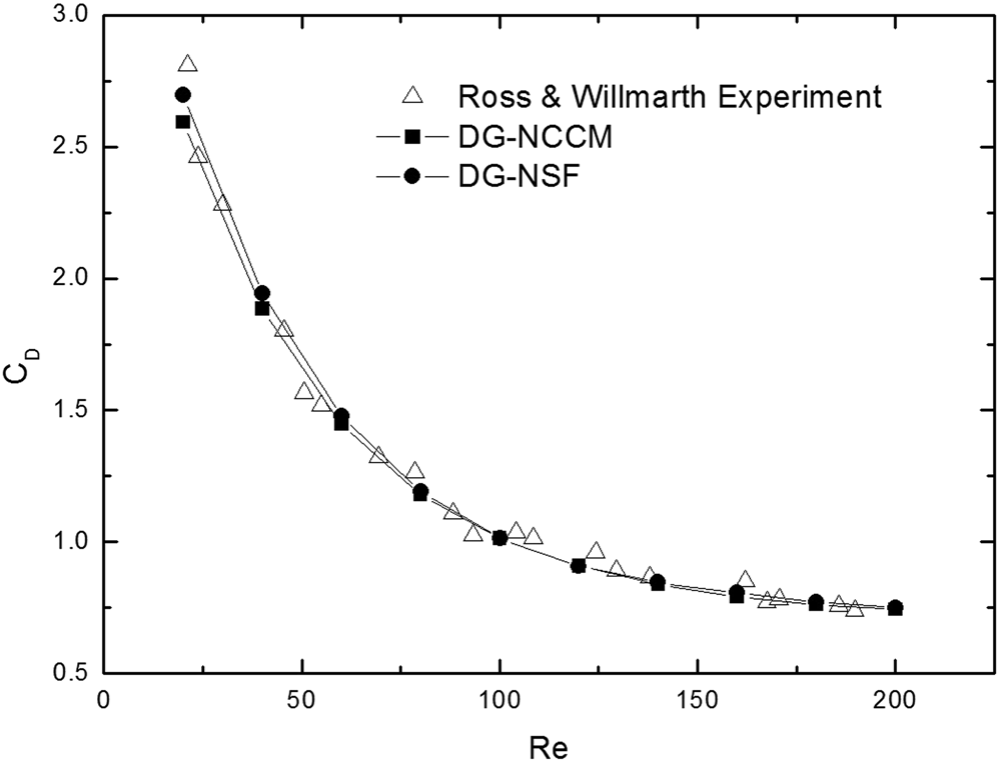
Comparison of the drag coefficient on the flow over a sphere at Ma = 0.01.

Figure 9 denotes the drag coefficient of a sphere. It shows that the present DG-NCCM method has good agreement with that of DG-NSF, and with experiment. The only slight difference is observed in the cases of very low Reynolds numbers, less than 50, and this difference might result from numerical error. This study of the gas flow around a sphere in the low Reynolds number regime demonstrates the capability of the present DG-NCCM for continuum gas flow to some degree.

**Figure 10:**
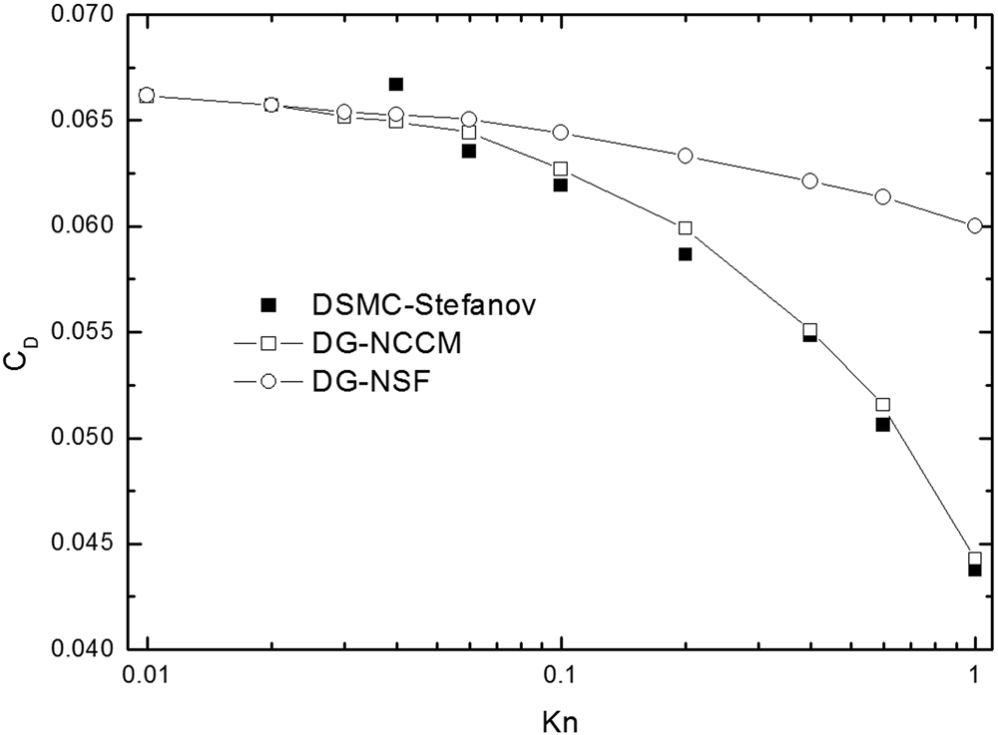
Normalized drag coefficient versus Knudsen number at Re = 0.125.

Low Reynolds number slip flow past a confined microsphere has been studied by Stefanov, Barber^40^, *et al*. The current study has investigated the use of two numerical approaches based upon continuum (NSF) and molecular (DSMC) model descriptions. and, considered two important fundamental questions. One is the upper limit of the Knudsen number for which a continuum slip-boundary solution (NSF) might be valid. Another is whether the DSMC method is able to cover a sufficiently wide range of Knudsen numbers so that continuum and molecular data can be compared. The simulations consider a fixed Reynolds number (*Re* = 0.125) and a range of Knudsen numbers, from *Kn* = 0.01 (continuum flow regime) to *Kn* = 1.0. We compare the drag coefficient on the microsphere, *C*_*D*_ versus the Knudsen number, as shown in Fig. 10. The DG-NSF calculations over-predict the drag coefficient at all high Knudsen numbers but converge to the DG-NCCM and DSMC solution as Kn tends to zero. This is also observed in Barber’s study^40^. The DG-NCCM simulations are found to be in good agreement with DSMC at high Knudsen numbers. In gas flows, the Knudsen number indicates whether the continuum hypothesis holds. Small Knudsen numbers represent the continuum regime, and high values represent rarefied gas flow. Generally, one part of the simulated domain can be described by solving the NSF equations for continuum fluid dynamics. For the other part, the density of particles is so low that this is described by a rarefied flow, which is usually simulated by DSMC. NCCM has a higher computational efficiency than does DSMC. Therefore, the present NCCM provides a better method that allows considerations of the whole flow regime.

### C. The hypersonic gas flow around Apollo 6 Command Module

**Figure 11:**
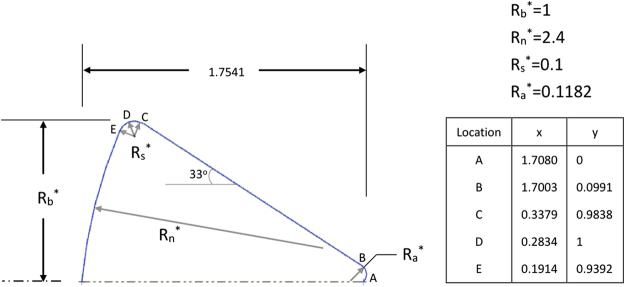
Apollo outer moldule line.

**Figure 12:**
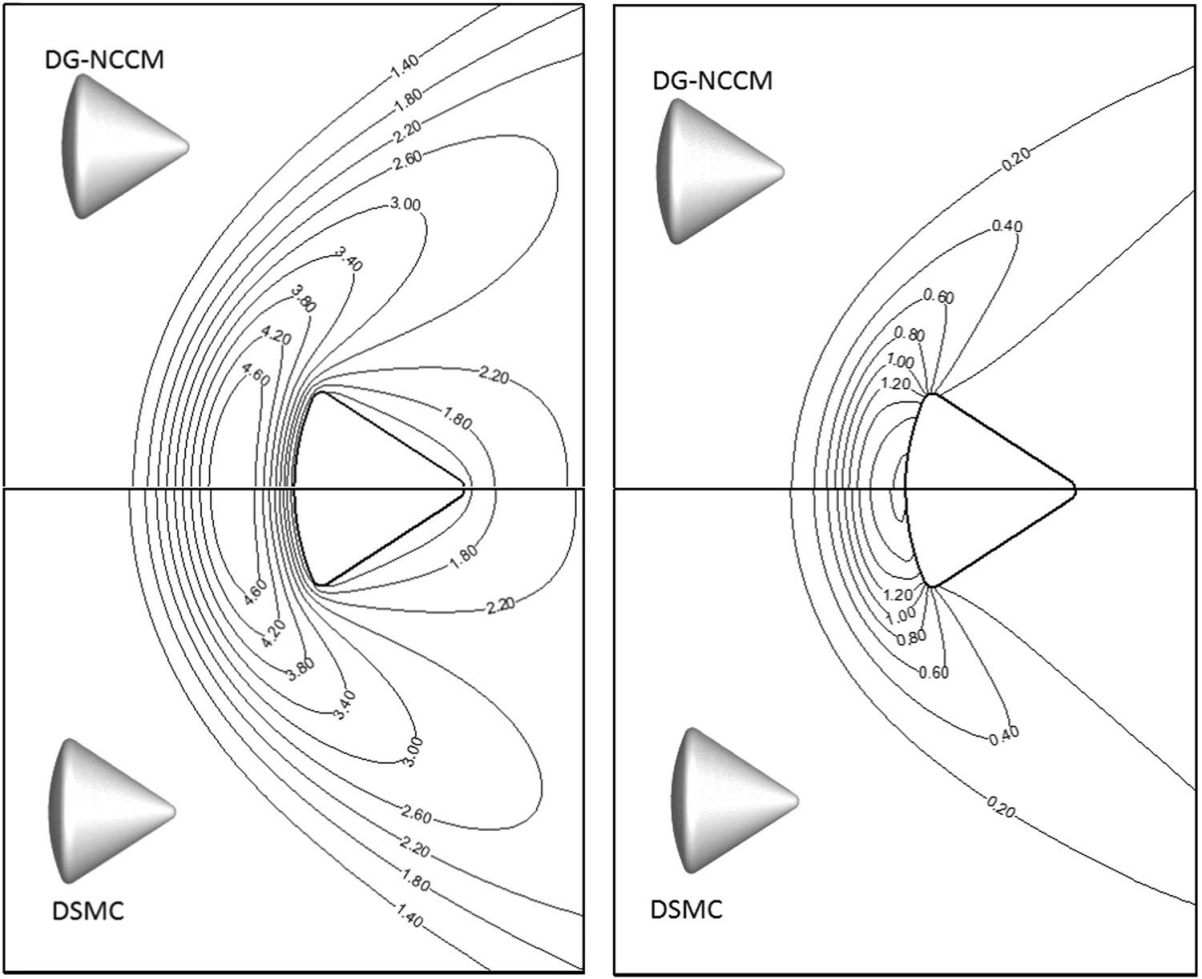
The comparisons of temperature and pressure coefficients at Ma = 5.0, Kn = 0.5.

**Figure 13:**
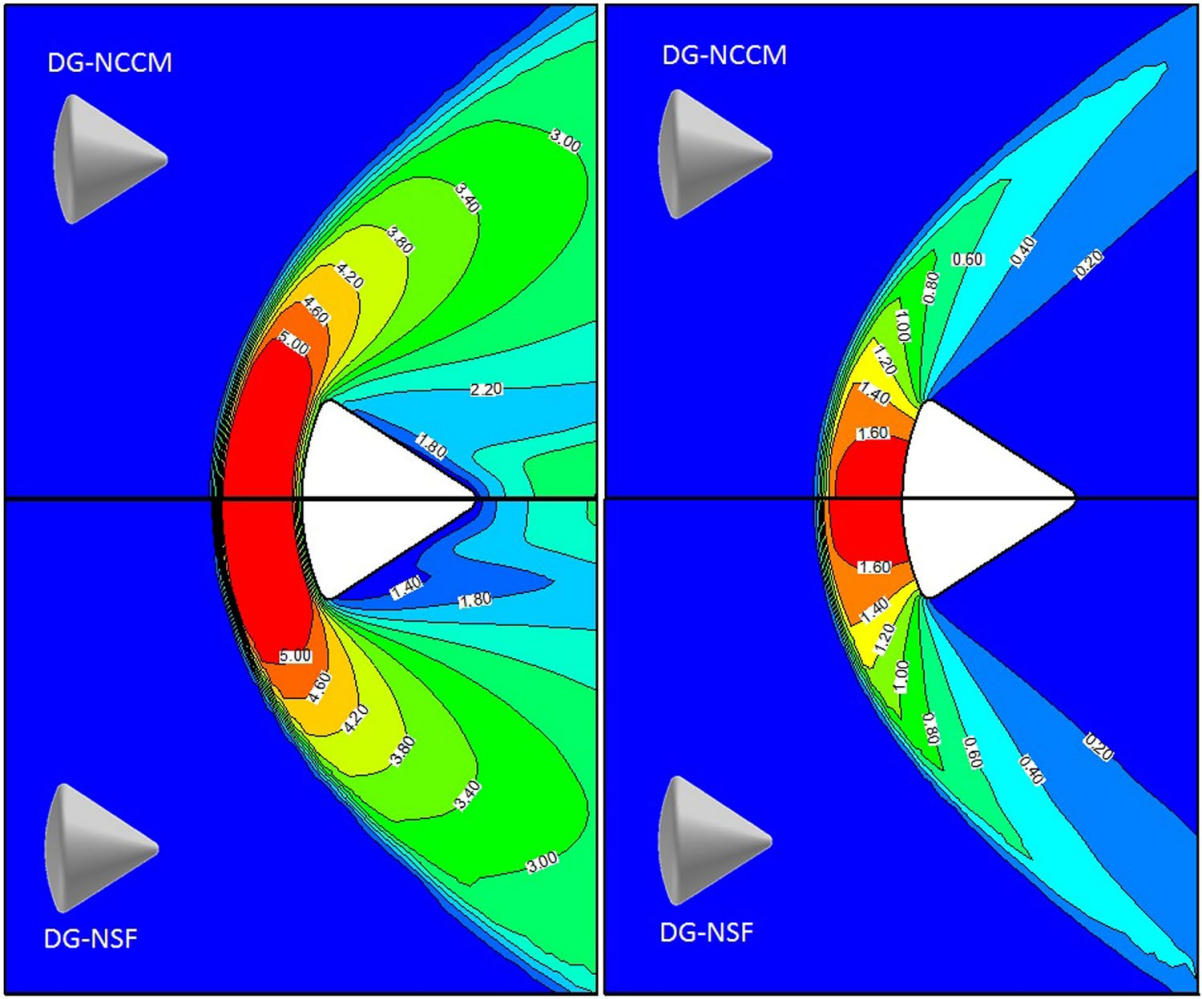
The comparisons of temperature and pressure coefficients at Ma = 5.0, Kn = 0.01.

In this section, we extend the present DG-NCCM scheme to more complex problems: the gas flows around the Apollo 6 command module^41^. The axisymmetric geometry used in the present study is shown in Fig. 11. The Apollo model is placed in the middle of a 30*d* × 30*d* sphere calculation domain, and *d* is the maximum diameter. We placed 5,229 triangle elements on the solid surface; the calculation domain consists of a total of 1,525,230 cells. Solutions were considered to have converged when the surface properties became steady and changed by less than 10^−7^ after additional integration cycles. The free stream atmospheric conditions details are given in Table 1. The surface temperature is assumed to be uniformly distributed at constant values and equal to that of the free stream. The Knudsen numbers are based on the free stream conditions and a characteristic length of 3.912 m (maximum capsule diameter). We performed DSMC simulation using the open software of Bird^6^, and the comparisons of temperature and pressure coefficients are shown in Fig. 12. Good agreements are observed except the slight difference in temperature distributions between the DG-NCCM and DSMC. Also, we compare the temperature and pressure coefficient contours for DG-NCCM and DG-NSF at *Ma* = 5.0 and *Kn* = 0.01, as shown in Fig. 13. The agreements are good, as expected.

**Table 1:** Free stream atmospheric conditions around the Apollo 6 Command Module.

*Ma*	*Kn*	*T*_0_/*K*	*T*_*w*_/*K*
5.0	0.5	273.0	273.0
5.0	0.01	273.0	273.0

## Discussion

In this study, we construct a numerical scheme to solve generalized hydrodynamic equations, named the Nonlinear Coupled Constitutive Model(NCCM), within the Galerkin framework. The main emphasis is on how to treat the complex highly nonlinear transport equations for non-conserved variables that arise from the high degree of thermal nonequilibrium in multi-dimensional gas flow situations. To the best knowledge of the authors, no numerical method for Nonlinear Coupled Constitutive Model has been reported in the literature. The present study may be regarded as the first computational attempt at solving the Nonlinear Coupled Constitutive Model to investigate both equilibrium and nonequilibrium gas flows.

For verification and validation, we apply the present scheme to a stiff problem of hypersonic gas flows around a 2D cylinder, a 3D sphere and an Apollo configuration both in the continuous and rarefied states. Then, we compare the results from the present DG-NCCM with those from DG-NSF and DSMC in continuum and rarefied states. The numerical results show that the present DG-NCCM scheme yields solutions in better agreement with the DSMC scheme and the experimental data than are the DG-NSF results, in all studied cases of rarefied problems. Furthermore, good agreement is observed between the DG-NCCM and DG-NSF results in the continuum cases. This findings indicate that the present study provides a unified framework for modeling continuum and rarefied gas flows.

## Electronic supplementary material

Supplementary material
