# Supplementary material for: A Unified Framework for Modeling Continuum and Rarefied Gas Flows
Source: Sci Rep. 2017 Oct 12;7:13108. doi: 10.1038/s41598-017-13274-7 (PMC5638930; doi:10.1038/s41598-017-13274-7)
Supplement: Supplementary file 1 — Supplementary material [file 41598_2017_13274_MOESM1_ESM.pdf]

A Unified Framework for Modeling Continuum and Rarefied Gas Flows

Hong Xiao[1,2,*] **and Ke Tang**[1,+]

[1]School of Power and Energy, Northwestern Polytechnical University, Xi'an, 710072, China
[2]Department of Engineering, University of Cambridge, Cambridge, CB2 1PZ, UK
[*]xhong@nwpu.edu.cn, hx251@cam.ac.uk
[+]these authors contributed equally to this work

ABSTRACT

This is the appendices of ”A Unified Framework for Modeling Continuum and Rarefied Gas Flows”.

Although some studies have previously been reported by Eu, Myong and Xiao[1–4], we review the detailed process in the following section to facilate understanding. Also the further references can be found on pages of 365-397 in Eu' Book[1], and pages of 182-183 in Xiao and Myong' paper[4].

The Boltzmann-Curtiss equation, at the molecular level, can be expressed as (in the absence of external forces for gas),

$$\left[\frac{\partial}{\partial t}+\mathbf{v}\cdot\nabla+\frac{j}{I}\frac{\partial}{\partial\psi}\right]f(\mathbf{v},\mathbf{r},t)=C[f] \tag{1}$$

where the term $C[f]$ represents the collision integral of the interaction among the particles.

A. Conservation Laws

The Boltzmann-Curtiss equation fulfils the requirements of the mass, momentum and energy conservation laws. By differentiating the densities of mass, momentum and energy, and omitting external force, simplified conservation equations can be obtained as follows[1,5]:

$$\begin{aligned}
&\frac{\partial\rho}{\partial t}+\nabla\cdot\rho\mathbf{u}=0\\
&\frac{\partial\rho\mathbf{u}}{\partial t}+\nabla\cdot(\rho\mathbf{u}\mathbf{u}+\mathbf{P})=0\\
&\frac{\partial\rho E}{\partial t}+\nabla\cdot(\mathbf{Q}+\rho\mathbf{u}E)+\mathbf{P}:\nabla\mathbf{u}=0
\end{aligned} \tag{2}$$

$\mathbf{P}$ and $\mathbf{Q}$ are non-conservation variables whose molecular expression does not yield a collisional invariant.

B.Velocity Moment

To derive the evolution equations for nonconservation variables, we need to define the velocity moment of order l for component i,

$$\mathbb{R}_i^{abc...l}=\left\langle m_i\mathbf{c}_{ia}\mathbf{c}_{ib}\mathbf{c}_{ic}...\mathbf{c}_{il}f_i(\mathbf{v}_i,\mathbf{r},t)\right\rangle \tag{3}$$

Then, differencing it with time,

$$\frac{\partial}{\partial t}\mathbb{R}_i^{abc...kl}=-\partial_t u_a\mathbb{R}_i^{bc...l}-\partial_t u_b\mathbb{R}_i^{ac...l}-\partial_t u_c\mathbb{R}_i^{abd...l}-...-\partial_t u_l\mathbb{R}_i^{abc...k}+\left\langle m_i\mathbf{c}_{ia}\mathbf{c}_{ib}\mathbf{c}_{ic}...\mathbf{c}_{il}\partial_t f_i\right\rangle \tag{4}$$

Then, evolving the last collision term of above Eq. 4:

$$\begin{aligned}
S_{ab...kl}=\left\langle m_i\mathbf{c}_{ia}\mathbf{c}_{ib}\mathbf{c}_{ic}...\mathbf{c}_{il}\partial_t f_i\right\rangle&=\left\langle m_i\mathbf{c}_{ia}\mathbf{c}_{ib}\mathbf{c}_{ic}...\mathbf{c}_{ili}\Big(\sum_{j=1}^{r}C(f_i,f_j)-\mathbf{v}_i\cdot\nabla f_i-\mathbf{F}_I\cdot\nabla_{v_i}f_i\Big)\right\rangle\\
&=-(\mathbf{u}\cdot\nabla u_a)\mathbb{R}_i^{bc...l}-(\mathbf{u}\cdot\nabla u_b)\mathbb{R}_i^{ac...l}-(\mathbf{u}\cdot\nabla u_c)\mathbb{R}_i^{abd...l}-...-(\mathbf{u}\cdot\nabla u_l)\mathbb{R}_i^{ab...k}\\
&\quad-\mathbb{R}_i^{\cdot bc...l}\cdot\nabla u_a-\mathbb{R}_i^{a\cdot c...l}\cdot\nabla u_b-\mathbb{R}_i^{ab\cdot d...l}\cdot\nabla u_c-...-\mathbb{R}_i^{abc...k}\cdot\nabla u_l\\
&\quad+F_{ia}\mathbb{R}_i^{bc...l}+F_{ib}\mathbb{R}_i^{ac...l}+F_{ic}\mathbb{R}_i^{abd...l}+...+F_{il}\mathbb{R}_i^{abc...k}\\
&\quad-\nabla\cdot(\mathbb{R}_i^{\cdot ab...l}+\mathbf{u}\mathbb{R}_i^{abc...l})+\Lambda_i^{(\mathbb{R})(abc...kl)}
\end{aligned} \tag{5}$$

Here,

$$\Lambda_i^{(\mathbb{R})(abc...kl)}=\sum_{j-1}^{r}\left\langle m_i\mathbf{c}_{ia}\mathbf{c}_{ib}\mathbf{c}_{ic}...\mathbf{c}_{il}C(f_i,f_j)\right\rangle$$

Finally, the derivative of the velocity moment can be obtained,

$$
\frac{\partial}{\partial t}\mathbb{R}_i^{abc\ldots kl} = -\nabla\cdot(\mathbb{R}_i^{\cdot ab\ldots l} + \mathbf{u}\mathbb{R}_i^{abc\ldots l}) - \sum_{all}\left(\frac{d\mathbf{u}}{dt}\right)_a \mathbb{R}_i^{bc\ldots l} - \sum_{all}\mathbb{R}_i^{\cdot bc\ldots l}\cdot\nabla u_a
$$

$$
+\sum_{all} F_{ia}\mathbb{R}_i^{bc\ldots l} + \Lambda_i^{(\mathbb{R})(abc\ldots kl)} \tag{6}
$$

Implementing the stress tensor and heat conductivity into Eq. 6,

$$
\mathbf{P} = \sum_{i=1}^{r}\left\langle m_i\mathbf{c}_i\mathbf{c}_i f_i(\mathbf{v}_i,\mathbf{r};t)\right\rangle \qquad \mathbf{Q} = \sum_{i=1}^{r}\mathbf{Q}_i = \sum_{i=1}^{r}\left\langle \frac{1}{2}m_i c_i^2\mathbf{c}_i f_i(\mathbf{v}_i,\mathbf{r};t)\right\rangle
$$

We can also obtain the transport equation for the stress tensor and heat conductivity,

$$
\frac{\partial}{\partial t}\mathbf{P}_i = -\nabla\cdot(\varphi_i^{(3)} + \mathbf{u}\mathbf{P}_i) - (d_t\mathbf{u}\mathbf{J}_i + \mathbf{J}_i d_t\mathbf{u}) - \left[\mathbf{P}_i\cdot\nabla\mathbf{u} + (\nabla\mathbf{u})^t\cdot\mathbf{P}_i\right] + (\mathbf{F}_i\mathbf{J}_i + \mathbf{J}_i\mathbf{F}_i) + \Lambda_i^{(P)}
$$

$$
\frac{\partial}{\partial t}\mathbf{Q}_i = -\nabla\cdot(\varphi_i^{(4)} + \mathbf{u}\mathbf{Q}_i) - d_t\mathbf{u}\cdot(\mathbf{P}_i + \rho_i\mathbf{c}_i\mathbf{U}) - \varphi_i^{(3)} : \nabla\mathbf{u} + \mathbf{Q}_i\cdot\nabla\mathbf{u} + \mathbf{F}_i(\mathbf{P}_i + \rho_i\mathbf{c}_i\mathbf{U}) + \Lambda_i^{(Q)} \tag{7}
$$

Here

$$
\varphi_i^{(3)} = < m_i\mathbf{c}_i\mathbf{c}_i\mathbf{c}_i f_i >, \varphi_i^{(4)} = \left\langle\frac{1}{2}m_i\mathbf{c}_i^2\mathbf{c}_i\mathbf{c}_i f_i\right\rangle, \Lambda_i^{(P)} = \sum_{j=1}^{r}\left\langle m_i\mathbf{c}_i\mathbf{c}_i C(f_i,f_j)\right\rangle, \Lambda_i^{(Q)} = \sum_{j=1}^{r}\left\langle\frac{1}{2}m_i c_i^2\mathbf{c}_i C(f_i,f_j)\right\rangle \tag{8}
$$

It can be seen that there are four terms in these transport equations: derivation with time, a gradient of high-order terms, and flux and collision parts.

C. The definition of distribution function

The definition of the distribution function is expressed via Eu Methods[1] as follows:

$$
f_i = exp\left[-\frac{1}{k_B T}(H_i + H_i^{(1)} - \mu_i)\right] \tag{9}
$$

In this equation,

$$
H_i = \frac{1}{2}m_i\mathbf{c}_i^2 + m_i\Psi_i(r), H_i^{(1)} = \sum X_i^{(a)}\odot h_i^{(a)}, exp\left(-\frac{1}{k_B T}\mu_i\right) = n_i^{-1}\left\langle exp\left[-\frac{1}{k_B T}(H_i + H_i^{(1)})\right]\right\rangle
$$

Here, H_i can be regarded as the local equilibrium, $H_i^{(1)}$ denotes the nonequilibrium condition, μ_i connects the local equilibrium and nonequilibrium conditions to fulfil the conservation laws and the positive entropy generation, $\mathbf{c}_i = \mathbf{v}_i - \mathbf{u}$ denotes thermal motion velocity, $\Psi_i(r)$ denotes potential energy, $h^{(a)}$ is the molecular expression for moments of the nonequilibrium variable, X_a denotes a weighting coefficient.

If we substitute this definition of the distribution function into the entropy balance equation, the entropy generation can be obtained:

$$
\sigma_{ent} = \frac{1}{T}\sum_{i=1}^{r}\sum X_i^{(a)}\odot\Lambda^{(a)} \geqslant 0 \tag{10}
$$

This form renders a clear, physical interpretation of entropy production: the entropy production is a direct measure of the energy dissipation arising from molecular collisions in the system, which in turn gives rise to dissipative evolution of nonconserved macroscopic fluxes (moments).

In addition, the local equilibrium distribution function can be given in a form similar to the exponential form as follows:

$$
f_i^0 = exp[-\beta(H_i - \mu_i^0)], exp(\beta\mu_i^0) = n_i^{-1} < exp(-\beta H_i) > \tag{11}
$$

By applying the following dimensionless processes,

$$\bar{\mu}_i = \mu_i/T, \Delta\bar{\mu}_i = \bar{\mu}_i - \bar{\mu}_i^0, \bar{X}_i^{(a)} = X_i^{(a)}/T \tag{12}$$

the local equilibrium distribution function can be further written as,

$$f_i = f_i^{(0)} exp\big[-k_B^{-1}\big(\sum \bar{X}_i^{(a)} \odot h_i^{(a)} - m_i\Delta\bar{\mu}_i\big)\big] \tag{13}$$

Moreover, the molecular expressions of the viscous stress, excess normal stress and heat conductivity can be rewritten again.

The unknown functions of $X_i^{(a)}$ now must be calculated in terms of the macroscopic variables obeying the evolution equations of mass, momentum and energy (conservation laws). We substitute the nonequilibrium canonical form of the distribution function into the Boltzmann equation and multiply by $h_i^{(a)}$ to obtain the energy dissipation:

$$\left\langle f_i h_i^{\gamma}(d_t + \mathbf{c}\cdot\nabla + \mathbf{F}_i\cdot\nabla_{vi})(k_B^{-1})(\sum_a \bar{X}_i^{(a)} \odot h_i^{(a)} - m_i\Delta\bar{\mu}_i)\right\rangle = \Lambda_i^{(\gamma)} \tag{14}$$

In this equation, $\Lambda_i^{(\gamma)}$ denotes the energy dissipation and represents the collision term of the transportation equation for non-conservation variables.

For comparison of $\Lambda_i^{(\gamma)}$, we define

$$\hbar_i^{(a)} = h_i^{(a)}\left[1 + k_B^{-1}\sum_a \bar{X}_i^{(a)} \odot h_i^{(a)} - m_i\Delta\bar{\mu}_i\right] \tag{15}$$

Consider the transport equation of $\langle f_i\hbar_i^{(\gamma)}\rangle$:

$$\rho d_t\big(\rho^{-1}\langle f_i\hbar_i^{(\gamma)}\rangle\big) = -\nabla\cdot\langle\mathbf{c}_i f_i\hbar_i^{(\gamma)}\rangle + \langle f_i(d_t + \mathbf{c}_i\cdot\nabla + \mathbf{F}_i\cdot\nabla_{vi})\hbar_i^{(\gamma)}\rangle + \sum_{j=1}^{r}\langle\hbar_i^{(\gamma)}C(f_i,f_j)\rangle \tag{16}$$

Comparing this equation with the transportation equation for non-conservation variables, it can be rewritten as

$$\rho d_t\big(\rho^{-1}\langle f_i h_i^{(\gamma)}\rangle\big) = -\nabla\cdot\langle\mathbf{c}_i f_i h_i^{(\gamma)}\rangle + \langle f_i(d_t + \mathbf{c}_i\cdot\nabla + \mathbf{F}_i\cdot\nabla_{vi})h_i^{(\gamma)}\rangle + \sum_{j=1}^{r}\langle h_i^{(\gamma)}C(f_i,f_j)\rangle \tag{17}$$

equation (16) is equivalent to equation (17), leading to the conclusion that $\langle f_i\hbar_i^{(\gamma)}\rangle$ must be a constant multiple of $\Phi_i^{(\gamma)}$:

$$\langle f_i\hbar_i^{(\gamma)}\rangle = b\langle f_i h_i^{(\gamma)}\rangle = b\Phi_i^{(\gamma)} \tag{18}$$

Here, b is a constant. Equations (14) and (17) are both the transport equations of non-conservation variables coupled with the Boltzmann equation, with the distinction being (16); thus, $b = 0$, and we have

$$\sum_a\langle f_i h_i^{(\gamma)} h_i^{(a)}\rangle \odot \bar{X}_i^{(a)} = -(k_B - m_i\Delta\bar{\mu}_i)\Phi_i^{(\gamma)} \tag{19}$$

In the lowest-order approximation of the dissipation term, by applying the model of small perturbation, we can set $f_i = f_i^0$ and $\Delta\bar{\mu}_i = 0$. Thus,

$$\sum_a\langle f_i^0 h_i^{(\gamma)} h_i^{(a)}\rangle \odot \bar{X}_i^{(a)} = -k_B T\Phi_i^{(\gamma)} \tag{20}$$

The moments of $h_i^{(a)}$ are treated as orthogonal tensor Hermit polynomials; as a result, we have

$$\langle f_i^0 h_i^{(\gamma)} h_i^{(a)}\rangle = \delta_{a\gamma}\langle f_i^0 h_i^{(\gamma)} h_i^{(\gamma)}\rangle \tag{21}$$

Finally, we obtain

$$\langle f_i^0 h_i^{(\gamma)} h_i^{(\gamma)}\rangle \odot \bar{X}_i^{(\gamma)} = -k_B T\Phi_i^{(\gamma)} \tag{22}$$

And

$$X_i^{(1)} = -\mathbf{\Pi}_i/2p_i, X_i^{(2)} = -3\Delta_i/2p_i, X_i^{(3)} = -\mathbf{Q}/p_i\hat{h}_i \tag{23}$$

D. Constitutive relations

Usually, we use the viscous stress and excess normal stress to replace the stress tensor. For this purpose, the following formulations should be defined in the molecular expressions for moments:

$$h_i^{(1)} = [m_i \mathbf{c}_i \mathbf{c}_i]^{(2)}, h_i^{(2)} = \frac{1}{3} m_i c_i^2 - p_i/n, h_i^{(3)} = (\frac{1}{2} m_i c_i^2 + m_i \Psi_i - \hat{h} m_i)\mathbf{c}_i \tag{24}$$

When averaged over velocity, they yields the traceless part of the stress tensor, and we denote them by the symbol $\Phi_i^{(a)}$

$$\Phi_i^{(a)} = \left\langle h_i^{(a)} f_i(\mathbf{v}_i, \mathbf{r}; t) \right\rangle \tag{25}$$

where $a = 1,2,3$ and $i = 1,2,3,...,n$. The meaning of leading $\Phi_i^{(a)}$ is as follows:

$$\Phi_i^{(1)} = \mathbf{\Pi}_i = [\mathbf{P}_i]^{(2)}, \Phi_i^{(2)} = \Delta_i = \frac{1}{3} Tr P_i - p_i, \Phi_i^{(3)} = \mathbf{Q}_i \tag{26}$$

The transport equation of non-conservation variables can be obtained,

$$\rho \frac{d(\Phi_i^{(a)}/\rho)}{dt} + \nabla \cdot \psi^{(a)} = \Lambda_k + Z_k \tag{27}$$

Here, Λ_k denotes the collision term and can be expressed as:

$$\Lambda_k = \sum_{j=1}^{r} \left\langle h_i^{(a)} C(f_i, f_j) \right\rangle \tag{28}$$

E. Treatment of the Collision Term

First, we give the expression of entropy production

$$\begin{aligned} \sigma_{ent}(\mathbf{r}, t) &= -k_B \sum_{i=1}^{r} \sum_{j=1}^{r} \left\langle ln f_i C(f_i, f_j) \right\rangle \\ &= \frac{1}{4} k_B \sum_{i=1}^{r} \sum_{j=1}^{r} \int d\mathbf{v}_i \int d\mathbf{v}_j \int_0^{2\pi} d\phi \times \int_0^{\infty} ln(f_i^* f_j^* / f_i f_j)(f_i^* f_j^* - f_i f_j) g_{ij} b db \geqslant 0 \end{aligned} \tag{29}$$

By applying equation 13, the entropy production can be expressed as:

$$\sigma_{ent}(\mathbf{r}, t) = \frac{1}{4} k_B \sum_{i=1}^{r} \sum_{j=1}^{r} \int d\mathbf{v}_i \int d\mathbf{v}_j \int_0^{2\pi} d\phi \times \int_0^{\infty} f_i^{(0)} f_j^{(0)} \times [exp(-y_{ij}) - exp(-x_{ij})](x_{ij} - y_{ij}) g_{ij} b db \tag{30}$$

Here: $x_{i,j} = x_i + x_j, y_{i,j} = x_i^* + x_j^*$. We normalize entropy production,

$$\begin{aligned} \hat{\sigma}_{ent} &= \sigma_{ent} g / k_B, g = (m/2k_B T)^{1/2} / n^2 d^2 \\ \hat{\sigma}_{ent}(\mathbf{r}, t) &= \frac{1}{4} \sum_{i=1}^{r} \sum_{j=1}^{r} \left\langle \left\langle [exp(-y_{ij}) - exp(-x_{ij})](x_{ij} - y_{ij}) \right\rangle \right\rangle \end{aligned} \tag{31}$$

also, we define,

$$\begin{aligned} \kappa &= \frac{1}{2} \left\langle \left\langle \sum_{i=1}^{r} \sum_{j=1}^{r} (x_{ij} - y_{ij})^2 \right\rangle \right\rangle^{1/2} \\ \kappa_2 &= \frac{1}{4} \left\langle \left\langle \sum_{i=1}^{r} \sum_{j=1}^{r} (x_{ij} - y_{ij})^2 (x_{ij} + y_{ij}) \right\rangle \right\rangle \\ \kappa_3 &= \frac{1}{4} \left\langle \left\langle \sum_{i=1}^{r} \sum_{j=1}^{r} (x_{ij} - y_{ij})^2 (x_{ij}^2 + x_{ij} y_{ij} + y_{ij}^2) \right\rangle \right\rangle \end{aligned} \tag{32}$$

Then, entropy production can be further expressed as,

$$\hat{\sigma}_{ent}(\mathbf{r},t) = \frac{\kappa}{2}\left\{\begin{array}{l} exp\Big[\kappa - \frac{1}{2}(\kappa_2/\kappa + \kappa^2) + \frac{1}{3!}(\kappa_3/\kappa + 3\kappa_2 + 2\kappa^3) + ...\Big] \\ -exp\Big[-\kappa - \frac{1}{2}(\kappa_2/\kappa - \kappa^2) - \frac{1}{3!}(\kappa_3/\kappa - 3\kappa_2 + 2\kappa^3) + ...\Big] \end{array}\right\} \tag{33}$$

If we neglect the second or high-order terms, then,

$$\hat{\sigma}_{ent}(\mathbf{r},t) = \kappa sinh\kappa = \kappa^2 q(\kappa) = \kappa\frac{e^{\kappa} - e^{-\kappa}}{2} \tag{34}$$

When the gas flows approach the near equilibrium state from nonequilibrium, κ approaches to zero. Therefore, take the limitation,

$$\lim_{\kappa\to 0}(\hat{\sigma}_{ent}(\mathbf{r},t)) = \kappa^2 \tag{35}$$

And, the Rayleigh-Onsager dissipation function denotes the dissipation energy in near equilibrium states, so κ^2 is the Rayleigh-Onsager dissipation function.

$$\kappa = \frac{(mk_BT)^{1/4}}{\sqrt{2}pd}\left[\frac{\mathbf{\Pi}:\mathbf{\Pi}}{2\eta} + \gamma'\frac{\Delta^2}{\eta_b} + \frac{\mathbf{Q}\cdot\mathbf{Q}}{\lambda T}\right]^{1/2}. \tag{36}$$

When it approaches the near equilibrium state, $q(\kappa) = 1$. The nonlinear energy dissipation is limited to the linear Rayleigh-Onsager dissipation function of κ^2. Far from the equilibrium state, nonlinear factors $q(\kappa)$ gradually increase.

F. Hydrodynamic Equations

Finally, from Eqs. 2, 7, 27, 10 and 34, the new hydrodynamic equations can be rewritten as follows,

$$\frac{\partial \mathbf{U}}{\partial t} + \nabla\cdot\mathbf{F}_{\mathsf{inv}}(\mathbf{U}) + \nabla\cdot\mathbf{F}_{\mathsf{vis}}(\mathbf{U},\mathbf{\Pi},\Delta,\mathbf{Q}) = 0$$

$$\frac{\partial \mathbf{\Phi}}{\partial t} + \nabla\cdot(\mathbf{\Phi u}) + \Lambda_k + Z_k = 0$$

$$\mathbf{U} = \begin{pmatrix}\rho\\ \rho\mathbf{u}\\ \rho E\end{pmatrix}, \mathbf{F}_{\mathsf{inv}}(\mathbf{U}) = \begin{pmatrix}\rho\mathbf{u}\\ \rho\mathbf{uu} + p\mathbf{I}\\ (\rho E + p)\mathbf{u}\end{pmatrix}, \mathbf{F}_{\mathsf{vis}}(\mathbf{U},\mathbf{\Pi},\Delta,\mathbf{Q}) = \begin{pmatrix}0\\ \mathbf{\Pi} + \Delta\mathbf{I}\\ (\mathbf{\Pi} + \Delta\mathbf{I})\cdot\mathbf{u} + \mathbf{Q}\end{pmatrix} \tag{37}$$

$$\mathbf{\Phi} = \begin{pmatrix}\mathbf{\Pi}\\ \mathbf{\Pi} + \Delta\mathbf{I}\\ \mathbf{Q}\end{pmatrix}, Z_k = \begin{pmatrix}2(p+\Delta)[\nabla\mathbf{u}]^{(2)} + 2[\mathbf{\Pi}\cdot\nabla\mathbf{u}]^{(2)}\\ 2\gamma'(\mathbf{\Pi} + \Delta\mathbf{I}):\nabla\mathbf{u} + \frac{2}{3}\gamma' p\nabla\cdot\mathbf{u}\\ (p+\Delta)C_pT\nabla lnT + \mathbf{\Pi}\cdot C_p\nabla T + \mathbf{Q}\cdot\nabla\mathbf{u}\end{pmatrix}, \Lambda_k = \begin{pmatrix}\frac{p}{\eta}\mathbf{\Pi}q(\kappa)\\ \frac{2}{3}\gamma'\frac{p}{\eta_b}\Delta q(\kappa)\\ \frac{pC_p}{\lambda}\mathbf{Q}q(\kappa)\end{pmatrix}$$

References

1. Eu, B. C. Kinetic theory and irreversible thermodynamics. *NASA STI/Recon Tech. Rep. A* **93**, 24498 (1992).
2. Myong, R. A computational method for eu's generalized hydrodynamic equations of rarefied and microscale gasdynamics. *J. Comput. Phys.* **168**, 47–72 (2001).
3. Le, N., Xiao, H. & Myong, R. A triangular discontinuous galerkin method for non-newtonian implicit constitutive models of rarefied and microscale gases. *J. Comput. Phys.* **273**, 160–184 (2014).
4. Xiao, H. & Myong, R. Computational simulations of microscale shock–vortex interaction using a mixed discontinuous galerkin method. *Comput. & Fluids* **105**, 179–193 (2014).
5. Bird, G. *The DSMC method* (CreateSpace Independent Publishing Platform, 2013).
